# Ets-1 promoter-associated noncoding RNA regulates the NONO/ERG/Ets-1 axis to drive gastric cancer progression

**DOI:** 10.1038/s41388-018-0302-4

**Published:** 2018-05-18

**Authors:** Dan Li, Yajun Chen, Hong Mei, Wanju Jiao, Huajie Song, Lin Ye, Erhu Fang, Xiaojing Wang, Feng Yang, Kai Huang, Liduan Zheng, Qiangsong Tong

**Affiliations:** 1 Department of Surgery, Union Hospital, Tongji Medical College, Huazhong University of Science and Technology, 1277 Jiefang Avenue, 430022 Wuhan, Hubei Province China; 2 Department of Pathology, Union Hospital, Tongji Medical College, Huazhong University of Science and Technology, 1277 Jiefang Avenue, 430022 Wuhan, Hubei Province China; 3 Clinical Center of Human Genomic Research, Union Hospital, Tongji Medical College, Huazhong University of Science and Technology, 1277 Jiefang Avenue, 430022 Wuhan, Hubei Province China

## Abstract

Emerging studies have indicated the essential functions of long noncoding RNAs (lncRNAs) during cancer progression. However, whether lncRNAs contribute to the upregulation of v-ets erythroblastosis virus E26 oncogene homolog 1 (Ets-1), an established oncogenic protein facilitating tumor invasion and metastasis, in gastric cancer remains elusive. Herein, we identified *Ets-1* promoter-associated noncoding RNA (*pancEts-1*) as a novel lncRNA associated with the gastric cancer progression via mining of publicly available datasets and rapid amplification of cDNA ends. RNA pull-down, RNA immunoprecipitation, in vitro binding, and RNA electrophoretic mobility shift assays indicated the binding of *pancEts-1* to non-POU domain containing octamer binding (NONO) protein. Mechanistically, *pancEts-1* facilitated the physical interaction between NONO and Ets related gene (ERG), resulting in increased ERG transactivation and transcription of *Ets-1* associated with gastric cancer progression. In addition, *pancEts-1* facilitated the growth and aggressiveness of gastric cancer cells via interacting with NONO. In gastric cancer tissues, *pancEts-1*, *NONO*, and *ERG* were upregulated and significantly correlated with *Ets-1* levels. High levels of *pancEts-1*, *NONO*, *ERG*, or *Ets-1* were respectively associated with poor survival of gastric cancer patients, whereas simultaneous expression of all of them (HR = 3.012, *P* = 0.105) was not an independent prognostic factor for predicting clinical outcome. Overall, these results demonstrate that lncRNA *pancEts-1* exhibits oncogenic properties that drive the progression of gastric cancer via regulating the NONO/ERG/Ets-1 axis.

## Introduction

Gastric cancer is the second leading cause of cancer-related death around the world [1]. Despite recent progress in surgical and comprehensive therapies, the clinical consequence of gastric cancer patients suffering from tumor invasion and metastasis remains to be improved [1]. Thus, it has been a focus to investigate the mechanisms essential for the development and progression of gastric cancer [2]. V-ets erythroblastosis virus E26 oncogene homolog 1 (*Ets-1*), one transcription factor of E26 transformation-specific (Ets) family, is upregulated in many solid tumors, such as breast [3], cervical [4], colorectal [5], lung [6], and ovarian [7, 8] cancers, which is associated with tumor angiogenesis and lymph node or distant metastasis [9]. Ets-1 promotes the growth and metastasis of different cancer cell lines [9], while knockdown of *Ets-1* inhibits cell transformation [10] and reverses the multiple drug resistance of breast cancer cells [11]. Our previous evidence shows that *Ets-1* levels are elevated in gastric cancer, and knockdown of *Ets-1* inhibits the invasiveness and metastasis of gastric cancer cells [12, 13]. However, the mechanisms contributing to high expression of *Ets-1* in gastric cancer remain to be determined.

Emerging studies have revealed the crucial functions of long noncoding RNAs (lncRNAs) during the progression of gastric cancer [14–17]. For example, lncRNA urothelial cancer associated 1 (*UCA1*) is upregulated in gastric cancer tissues, and promotes the growth and cell cycle process through binding to enhancer of zeste 2 polycomb repressive complex 2 subunit (EZH2) and facilitating *cyclin D1* expression in gastric cancer cells [14]. Metastasis-associated lung adenocarcinoma transcript 1 (*MALAT1*) increases the tumorigenesis and metastasis of gastric cancer via facilitating vasculogenic mimicry and angiogenesis [15]. LncRNA *XLOC_010235* is over-expressed in gastric cancer, and promotes the metastasis by associating with snail family zinc finger 1 [16]. Meanwhile, tumor suppressive forkhead box F1 adjacent noncoding developmental regulatory RNA (*FENDRR*) is downregulated in gastric cancer tissues, and inhibits the aggressive behaviors of cancer cells [17]. Thus, it is currently necessary to further determine the roles of lncRNAs in gastric cancer progression.

In this study, through mining of public datasets and performing rapid amplification of cDNA ends (RACE), we identified *Ets-1* promoter-associated noncoding RNA (*pancEts-1*) as a novel 1395-nt lncRNA associated with poor survival of gastric cancer. Our evidence indicates, for the first time, that *pancEts-1* is upregulated in clinical tissues and cell lines of gastric cancer. In addition, *pancEts-1* interacts with non-POU domain containing octamer binding (NONO) protein to facilitate its binding to Ets related gene (*ERG*), resulting in increased transactivation of ERG, transcription of *Ets-1*, and promotion of the in vitro and in vivo growth and aggresiveness of gastric cancer cells, suggesting the crucial roles of *pancEts-1*/NONO/ERG/Ets-1 axis in gastric cancer progression.

## Results

### *pancEts-1* is a lncRNA associated with poor survival of gastric cancer

**Fig. 1:**
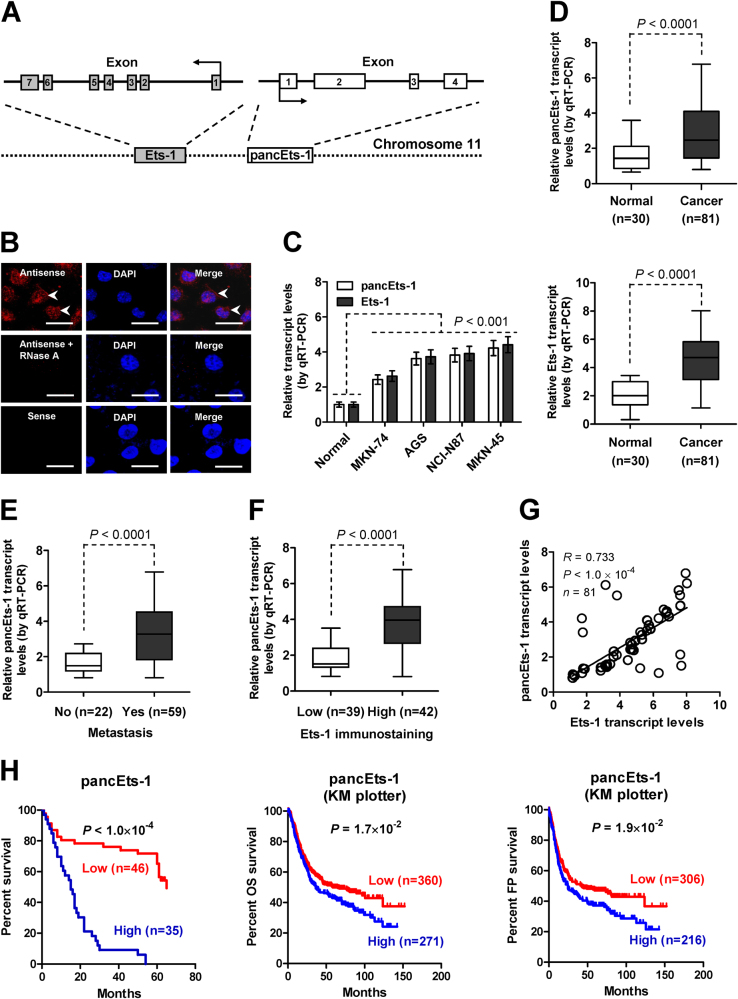
*pancEts-1* is a lncRNA associated with poor survival of gastric cancer. **a** Scheme indicating the existence of *pancEts-1* transcribed upstream the *Ets-1* promoter region. **b** RNA fluorescence in situ hybridization images showing the nuclear and cytoplasmic localization of *pancEts-1* in MKN-45 cells using a 138-bp antisense probe (red), with the nuclei staining by DAPI (blue). Sense probe and antisense probe with RNase A (20 μg) treatment were used as negative controls. Scale bars: 10 μm. **c** Real-time qRT-PCR assay revealing the *pancEts-1* transcript levels (normalized to β-actin) in normal gastric mucosa (*n* = 30) and cultured gastric cancer cell lines (mean ± SD, *n* = 5). **d** Real-time qRT-PCR assay indicating the differential expression of *pancEts-1* transcript (normalized to β-actin) in normal gastric mucosa (*n* = 30) and gastric cancer tissues (*n* = 81). **e**, **f** Real-time qRT-PCR assay showing the *pancEts-1* transcript levels (normalized to β-actin) in gastric cancer tissues with differential status of metastasis (**e**) or Ets-1 immunostaining (**f**). **g** The positive correlation between *pancEts-1* and *Ets-1* transcript levels in gastric cancer tissues (*n* = 81). **h** Kaplan–Meier curves indicating overall survival of 81 gastric cancer patients (cutoff value = 2.833) and overall (OS) and first progression (FP) survival of those derived from Kaplan-Meier plotter with low or high *pancEts-1* expression (cutoff values = 14.0 and 14.0). Student’s *t* test compared gene expression levels in **c**–**f**. Pearson’s correlation coefficient analysis in **g**. Log-rank test for survival comparison in **h**

Mining of UCSC Genome Browser revealed that *pancEts-1*, a novel lncRNA consisting of four exons, targeted the promoter region of *Ets-1* at chromosome 11q24.3 (Fig. 1a). The 5′-RACE and 3′-RACE analyses (Supplementary Figure S1A) revealed that *pancEts-1* was 1395 nucleotides (nt) in length and polyadenylated (GenBank Accession No. KP742344). Northern blot analysis confirmed the existence of 1.3-kb *pancEts-1* transcript in MKN-45 cells (Supplementary Figure S1B). Subcellular fractionation and RNA fluorescence in situ hybridization (RNA-FISH) assays revealed the nuclear and cytoplasmic enrichment as well as localization of *pancEts-1* in cultured gastric cancer cells (Supplementary Figure S1C and Fig. 1b). Meanwhile, the Coding Potential Assessment Tool (CPAT) [18] and Ribosome profiling data [19] revealed low protein-coding (probability value = 0.0103) or ribosome binding potential of *pancEts-1* (Supplementary Figure S1D). Mining of publicly available datasets indicated low frequency of mutation, deletion, or amplification of *pancEts-1* gene in human cancers (Supplementary Figure S1E). Real-time quantitative RT-PCR (qRT-PCR) revealed higher *pancEts-1* levels in cultured gastric cancer cells, when compared to those in normal gastric mucosa (Fig. 1c). We validated higher transcript levels of *pancEts-1* and *Ets-1* in an independent cohort of 81 primary gastric cancer specimens, than those in normal gastric mucosa (*P* < 0.0001, Fig. 1d). Higher *pancEts-1* levels were detected in gastric cancer cases with metastasis (*P* < 0.0001, Fig. 1e) or high Ets-1 immunostaining (*P* < 0.0001, Fig. 1f). Notably, the *pancEts-1* levels were positively correlated with those of *Ets-1* in gastric cancer specimens (*R* = 0.733, *P* < 1.0 × 10^-4^, Fig. 1g). In these 81 primary gastric cancer cases, Kaplan–Meier survival analysis indicated poor overall survival (*P* < 1.0 × 10^−4^) in patients with high *pancEts-1* expression (Fig. 1h). In addition, mining of public datasets derived from Kaplan-Meier plotter [20] and Gene Expression Omnibus (GEO) indicated that patients with high *pancEts-1* expression had lower overall (OS) and first progression (FP) survival possibility in gastric cancer, breast cancer, Ewing sarcoma, glioma, and lymphoma (Fig. 1h and Supplementary Figure S2). These data indicated that *pancEts-1*, a novel lncRNA, was associated with poor survival of gastric cancer.

### Knockdown of *pancEts-1* leads to suppression of gastric cancer progression

**Fig. 2:**
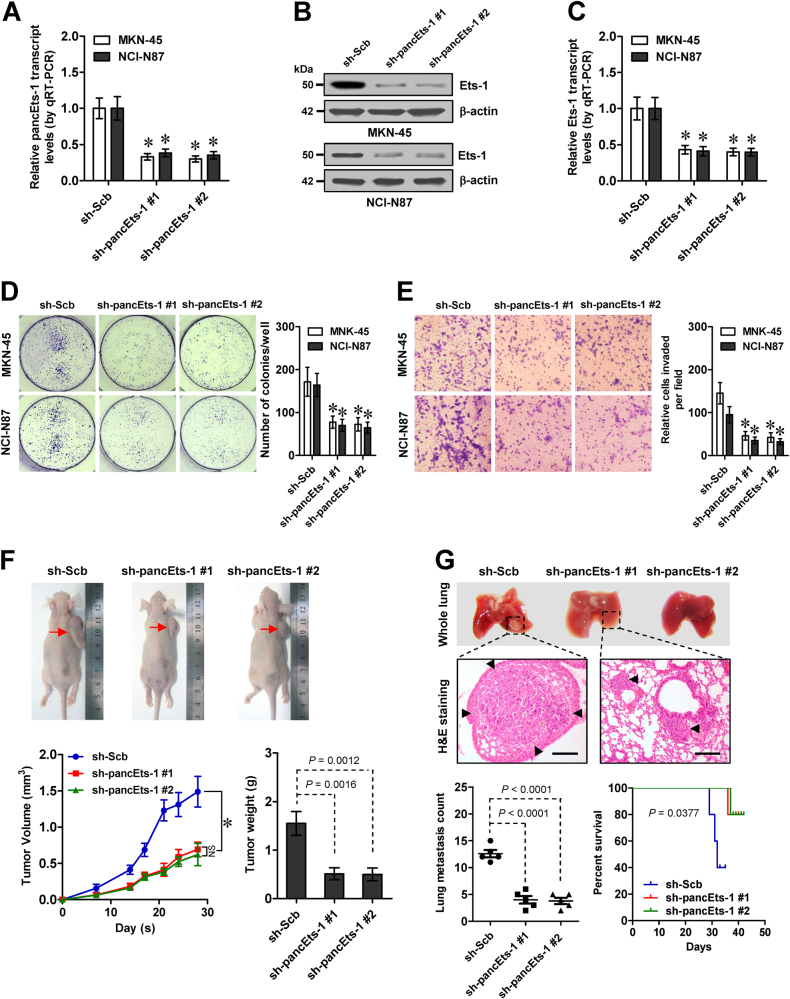
Knockdown of *pancEts-1* leads to suppression of gastric cancer progression. **a** Real-time qRT-PCR indicating the *pancEts-1* transcript levels (normalized to β-actin) in gastric cancer cells stably transfected with two independent shRNAs against *pancEts-1* (sh-pancEts-1 #1 and sh-pancEts-1 #2), compared with scramble shRNA (sh-Scb; mean ± SD, *n* = 5). **b**, **c** Western blot (**b**) and real-time qRT-PCR (**c**) showing the protein and transcript levels of *Ets-1* (normalized to β-actin) in MKN-45 and NCI-N87 cells stably transfected with sh-Scb, sh-pancEts-1 #1, or sh-pancEts-1 #2 (sh-Scb; mean ± SD, *n* = 5). **d**, **e** Colony formation (**d**) and transwell matrigel invasion (**e**) assays depicting the change in growth and invasiveness of gastric cancer cells stably transfected with sh-pancEts-1 #1 and sh-pancEts-1 #2, compared with sh-Scb (mean ± SD, *n* = 5). **f** Representative images (upper), in vivo growth curve (lower left), and tumor weight at the end points (lower right) of xenografts formed by subcutaneous injection of MKN-45 cells stably transfected with sh-Scb, sh-pancEts-1 #1, or sh-pancEts-1 #2 into the dorsal flanks of nude mice (*n* = 5 for each group). **g** Representative images (upper), HE staining (arrowheads, middle), and quantification (lower left) of lung metastatic colonization and Kaplan–Meier curves (lower right) of nude mice treated with tail vein injection of MKN-45 cells stably transfected with sh-Scb, sh-pancEts-1 #1, or sh-pancEts-1 #2 (*n* = 5 for each group). Student’s *t* test and analysis of variance compared the difference in **a** and **c**–**g**. Log-rank test for survival comparison in **g**. *P < 0.01 vs. sh-Scb. NS, not significant

Then, the functional impacts of *pancEts-1* knockdown were explored in gastric cancer cells representing high expression levels. Depletion of *pancEts-1* using two independent short hairpin RNAs (shRNAs), sh-pancEts-1 #1 and sh-pancEts-1 #2, in the MKN-45 and NCI-N87 cell lines led to a dramatic decrease in *Ets-1* expression levels (Fig. 2a–c). Stable knockdown of *pancEts-1* led to decrease in the viability (Supplementary Figure S3A), growth (Fig. 2d), and invasiveness (Fig. 2e) of MKN-45 and NCI-N87 cells in vitro. Consistent with these findings, the growth rate and tumor weight were significantly decreased in subcutaneous xenografts formed by MKN-45 cells with stable *pancEts-1* knockdown in nude mice (Fig. 2f). In addition, less metastatic colonies in the lung and improved survival were observed in nude mice received tail vein administration of gastric cancer cells with stable transfection of sh-pancEts-1 #1 or sh-pancEts-1 #2 (Fig. 2g). These data suggested that knockdown of *pancEts-1* inhibited the gastric cancer progression.

### *pancEts-1* interacts with NONO protein in cultured gastric cancer cells

**Fig. 3:**
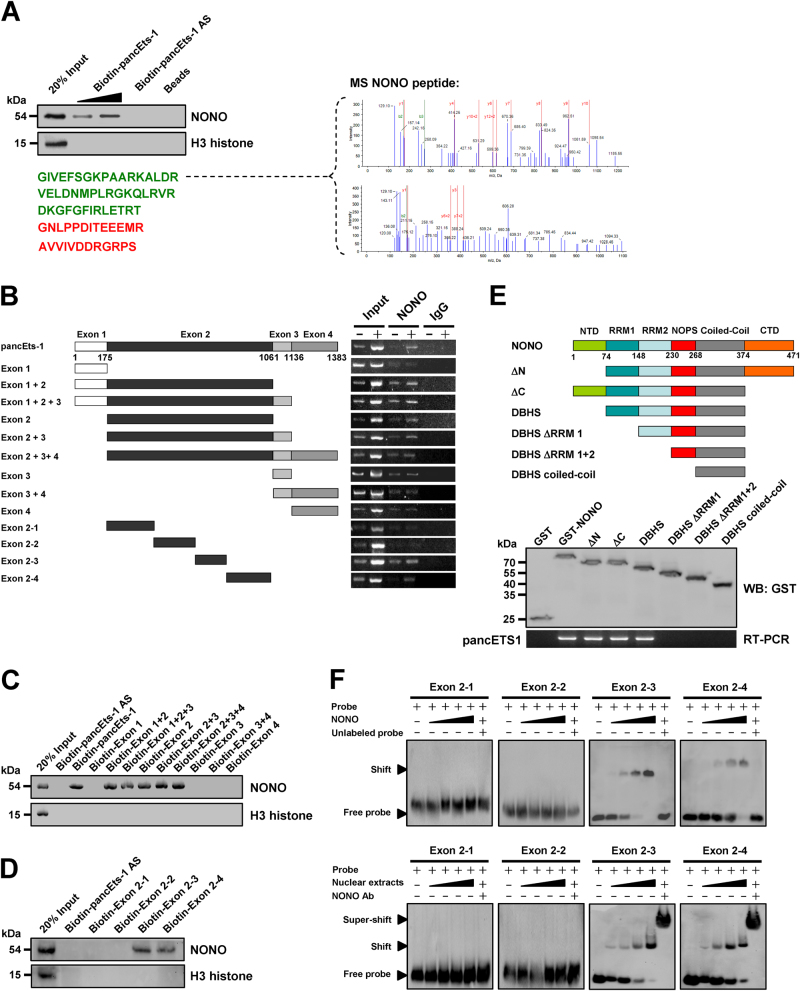
*pancEts-1* interacts with NONO protein in gastric cancer cells. **a** Biotin-labeled RNA pull-down (left) and mass spectrometry (MS) assay (right) showing the interaction between *pancEts-1* and NONO protein in MKN-45 cells. The *pancEts-1* antisense (AS)- and bead-bound protein served as negative controls. **b** RIP assay using NONO antibody indicating the interaction between *pancEts-1* and NONO protein in NCI-N87 cells transfected with a series of *pancEts-1* truncates. The IgG-bound RNA was taken as negative control. **c**, **d**, Western blot assay depicting the recovered NONO levels of cellular nuclear extracts pulled down by biotin-labeled *pancEts-1* truncates. **e** In vitro binding assay showing the recovered *pancEts-1* levels by RIP (lower) after incubation with full-length (1–471 amino acids), ΔN (75–471 amino acids), ΔC (1–374 amino acids), Drosophila behavior/human splicing (DBHS, 75–374 amino acids), ΔRRM1 (149–374 amino acids), ΔRRM1 + 2 (231–374 amino acids), or coiled-coil (269–374 amino acids) of GST-tagged recombinant NONO protein validated by western blot (upper). **f** RNA EMSA determining the interaction between recombinant or endogenous NONO protein and biotin-labeled RNA probes for *pancEts-1* (arrowhead), with or without competition using an excess of unlabeled homologous RNA probe or treatment using NONO antibody.

To determine the protein interacting with *pancEts-1*, we performed the biotin-labeled RNA pull-down and proteomic assays in MKN-45 cells. Mass spectrometry revealed that NONO, a nuclear RNA binding protein [21], was the protein with highest spectral counts (with 71 detectable peptides) binding to biotin-labeled *pancEts-1* (Fig. 3a). Western blot further confirmed the enrichment of NONO in RNA-protein complex pulled down by *pancEts-1*, but not in those pulled-down by beads only or *pancEts-1* antisense RNA (Fig. 3a). RNA immunoprecipitation (RIP) assay revealed an endogenous interaction between *pancEts-1* and NONO in MKN-45 and NCI-N87 cells (Supplementary Figure S3B). In addition, deletion-mapping analyses indicated the essential roles of exon 2 (especially the 691-1060 nt region) in interacting with NONO protein (Fig. 3b and Supplementary Figure S3C). Moreover, after incubation of cellular nuclear extracts with biotin-labeled *pancEts-1* truncates, RNA pull-down assay indicated that exon 2 (especially the 691-1060 nt region) of *pancEts-1* was able to interact with NONO protein (Fig. 3c, d). In vitro binding assay revealed that the RNA recognition motif 1 [RRM1, 75–148 amino acids (aa)], but not N-terminus (1–74 aa), RNA recognition motif 2 (RRM2, 149–230 aa), NonA/paraspeckle domain (NOPS, 231–268 aa), coiled-coil (269–374 aa), or C-terminus (375–471 aa), of glutathione S-transferase (GST)-tagged NONO protein was able to bind to *pancEts-1* (Fig. 3e). Consistently, RNA electrophoretic mobility shift assay (EMSA) indicated the capability of recombinant or endogenous NONO protein to bind to 691–1060 nt region of *pancEts-1*, which was recognized by NONO specific antibody (Fig. 3f). Meanwhile, treatment with competitive unlabeled homologous probe was able to completely ablate the formed complex (Fig. 3f). These results confirmed the specific binding of *pancEts-1* to NONO protein in gastric cancer cells.

### *pancEts-1* regulates Ets-1 expression by facilitating NONO-mediated ERG transactivation

**Fig. 4:**
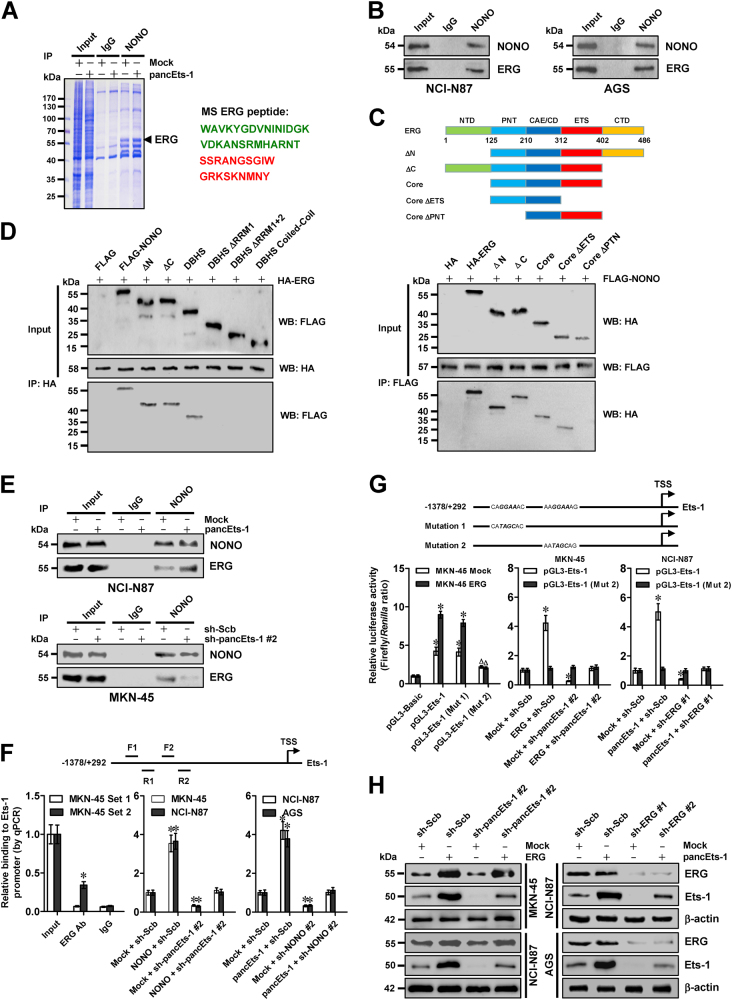
*pancEts-1* regulates Ets-1 expression by facilitating NONO-mediated ERG transactivation. **a** IP, Commassie blue staining (left) and mass spectrometry (MS) assay (right) showing the changes in NONO interacting proteins in NCI-N87 cells stably transfected with empty vector (mock) or *pancEts-1*. **b** IP and western blot revealing the endogenous interaction between NONO and ERG in the NCI-N87 and AGS cells, **c**, **d** IP and western blot indicating the interaction between NONO and ERG in NCI-N87 cells transfected with HA-tagged *ERG* or FLAG-tagged *NONO* truncates. **e** IP and western blot showing the interaction between NONO and ERG in NCI-N87 and MKN-45 cells stably transfected with mock, *pancEts-1*, scramble shRNA (sh-RNA), or sh-pancEts-1 #2. **f** ChIP and qPCR assays indicating the binding of ERG to *Ets-1* promoter in gastric cancer cells, and its changes in those stably transfected with mock, *NONO*, *pancEts-1*, sh-Scb, sh-NONO #2, or sh-pancEts-1 #2 (mean ± SD, *n* = 4). **g**, **h** Dual-luciferase (**g**) and western blot (**h**) assays showing the promoter activity and expression of *Ets-1* in gastric cancer cells, and their changes in those stably transfected with mock, *ERG*, *pancEts-1*, sh-Scb, sh-ERG #1, sh-ERG #2, or sh-pancEts-1 #2 (mean ± SD, *n* = 4). Student’s *t* test analyzed the difference in **f** and **g**. *P < 0.01 vs. IgG, mock + sh-Scb, or pGL3-Basic. ^Δ^*P* < 0.01 vs. pGL3-Ets-1

To identify the interacting transcription factor of NONO, we performed the co-immunoprecipitation (co-IP) and subsequent mass spectrometry assay, and found ERG as the NONO interacting protein facilitated by *pancEts-1* in NCI-N87 cells, with highest spectral counts (with 47 detected peptides; Fig. 4a). As shown in Fig. 4b and Supplementary Figure S3D, endogenous physical interaction between NONO and ERG was observed in NCI-N87 and AGS cells. To address their interaction domains, hemagglutinin (HA)-tagged *ERG* and FLAG-tagged *NONO* truncates were co-transfected into NCI-N87 cells. Co-IP and western blot assays demonstrated that the pointed domain (PNT, 126–210 aa), but not N-terminus (1–125 aa), central alternative exons/central domain (CAE/CD, 211–312 aa), ETS domain (313–402 aa), or C-terminus (403–486 aa), of ERG protein was essential for its binding to NONO (Fig. 4c). Meanwhile, RRM1 domain of FLAG-tagged NONO protein was able to interact with ERG (Fig. 4d). We then investigated the possible roles of *pancEts-1* in NONO-induced ERG transactivation, and found that ectopic expression or knockdown of *pancEts-1* respectively increased and decreased the interaction between NONO and ERG in NCI-N87 and MKN-45 cells (Fig. 4e). Notably, chromatin immunoprecipitation (ChIP) and real-time quantitative PCR (qPCR) revealed the binding of ERG to its target locus [−863/−724 bp upstream transcription start site (TSS)] in MKN-45 cells (Fig. 4f). As controls, no ERG enrichment on *Ets-1* promoter regions was observed in control samples immunoprecipitated by isotype immunoglobulin G (IgG) or measured by distant primer set (−1262/−1105 bp, Fig. 4f). Stable knockdown or ectopic expression of *pancEts-1* attenuated and facilitated the binding of ERG to *Ets-1* promoter in gastric cancer cell lines, which was rescued by transfection of *NONO* or sh-NONO #2, respectively (Fig. 4f). In dual-luciferase assay, the *Ets-1* promoter activity was facilitated and reduced by ectopic expression or knockdown of *ERG*, respectively (Fig. 4g), and mutation of ERG binding site 2, but not of ERG binding site 1, prevented these effects (Fig. 4g). Stable transfection of sh-pancEts-1 #2 or *pancEts-1* resulted in reduced and facilitated promoter activity and expression of *Ets-1*, which was rescued by over-expression or knockdown of *ERG*, respectively (Fig. 4g, h, and Supplementary Figure S3E). Collectively, these data indicated that *pancEts-1* regulated the Ets-1 expression by facilitating NONO-mediated ERG transactivation.

### *pancEts-1* harbors oncogenic properties through its interplay with NONO

**Fig. 5:**
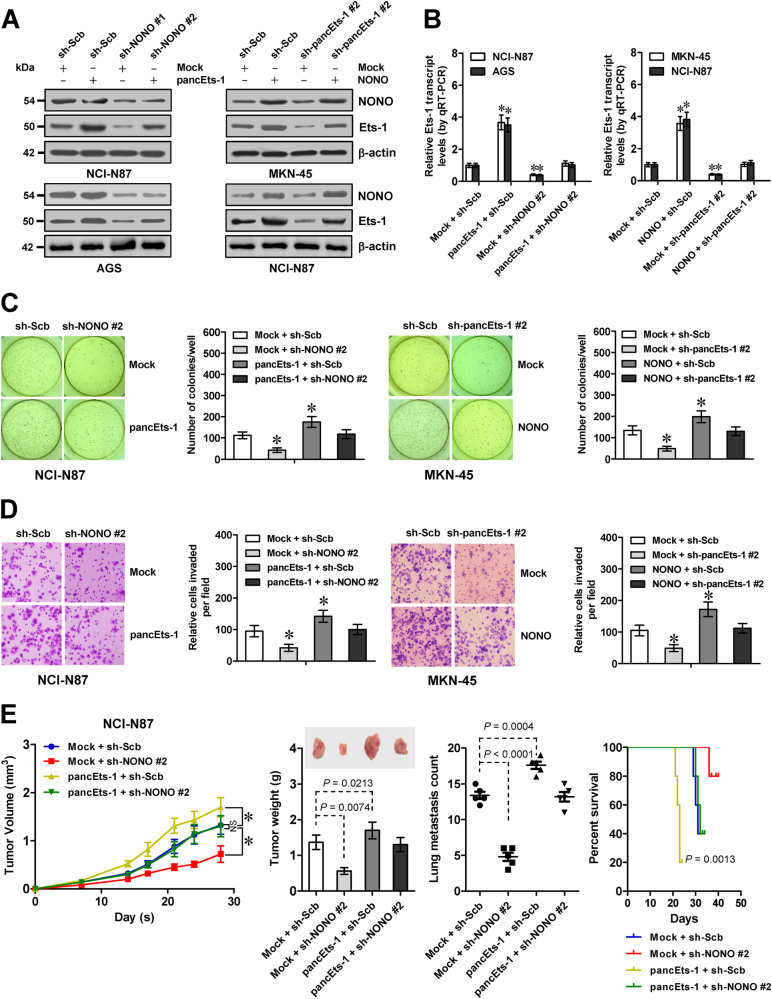
*pancEts-1* harbors oncogenic properties through its interplay with NONO. **a**, **b** Western blot (**a**) and real-time qRT-PCR (**b**) assays revealing the differential protein and transcript levels of *Ets-1* (normalized to β-actin) in gastric cancer cells stably transfected with mock, *pancEts-1*, *NONO*, sh-Scb, sh-NONO, or sh-pancEts-1 (mean ± SD, *n* = 4). **c** **d** Representative images (left) and quantification (right) of soft agar (**c**) and transwell matrigel invasion (**d**) assays indicating the anchorage-independent growth and invasion capability of gastric cancer cells stably transfected with mock, *pancEts-1*, *NONO*, sh-Scb, sh-NONO #2, or sh-pancEts-1 #2 (mean ± SD, *n* = 6). **e** In vivo growth curve (left), representative images (middle upper) and tumor weight (middle lower) at the end points of xenografts in athymic nude mice formed by hypodermic injection of NCI-N87 cells stably transfected with mock, *pancEts-1*, sh-Scb, and sh-NONO #2 (*n* = 5 for each group). Quantification of lung metastatic colonies (middle) and Kaplan–Meier curves (right) of nude mice treated with tail vein injection of NCI-N87 cells stably transfected with mock or *pancEts-1*, and those co-transfected with sh-Scb or sh-NONO #2 (*n* = 5 for each group). Student’s *t* test and analysis of variance analyzed the difference in **b**–**e** Log-rank test for survival comparison in **e**. *P < 0.01 vs. mock + sh-Scb. NS, not significant

To further investigate the roles of NONO in *pancEts-1*-mediated oncogenic properties, we performed rescue studies in cultured gastric cancer cell lines. Stable transfection of *pancEts-1* or sh-pancEts-1 #2 led to significantly increased and decreased *Ets-1* expression in gastric cancer cells (Fig. 5a, b), which was abolished by transfection of sh-NONO or *NONO*, respectively. In soft agar and matrigel invasion assays, stable ectopic expression or silencing of *pancEts-1* promoted and inhibited the anchorage-independent growth and invasiveness of gastric cancer cells, respectively (Fig. 5c, d). Transfection of sh-NONO #2 or *NONO* prevented the altered growth and invasiveness of gastric cancer cells with stable ectopic expression or silencing of *pancEts-1* (Fig. 5c, d). In mouse xenograft tumor assay, NCI-N87 cells stably transfected with *pancEts-1* displayed faster in vivo growth, and resulted in higher tumor weight of subcutaneous xenografts (Fig. 5e), more metastatic colonies in the lung (Fig. 5e), and shorter survival duration in athymic nude mice (Fig. 5e). Meanwhile, stable transfection of sh-NONO #2 abolished the impact of *pancEts-1* on in vivo growth and metastasis of NCI-N87 cells (Fig. 5e). These data indicated that *pancEts-1* harbored in vitro and in vivo oncogenic properties through its interplay with NONO.

### The expression of *NONO*, *ERG*, or *Ets-1* is associated with poor outcome of gastric cancer patients

**Fig. 6:**
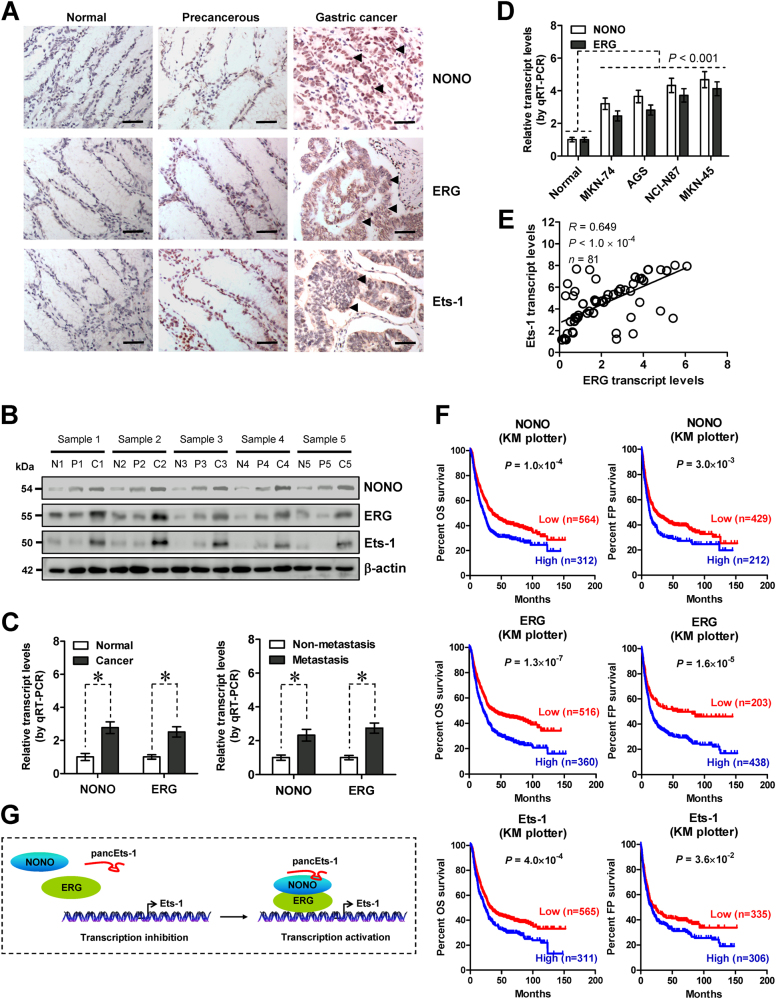
The expression of *NONO*, *ERG*, or *Ets-1* is associated with poor outcome of gastric cancer patients. **a** Representative immunohistochemical staining images showing the nuclear and cytoplasmic expression pattern of NONO, ERG, and Ets-1 in normal gastric mucosa, precancerous gastric mucosa, and tumor cells of gastric cancer specimens (arrowheads, brown). Scale bars: 100 μm. **b** Western blot assay indicating the differential levels of NONO, ERG, and Ets-1 in normal gastric mucosa (N, *n* = 30), precancerous tissues (P, *n* = 81), and gastric cancer tissues (**c**, *n* = 81). **c** Real-time qRT-PCR showing the expression levels (normalized to β-actin) of *NONO* and *ERG* in normal gastric mucosa (*n* = 30), gastric cancer tissues (*n* = 81), and gastric cancer specimens with (*n* = 22) or without metastasis (*n* = 59). **d** Real-time qRT-PCR assay revealing the *NONO* and *ERG* transcript levels (normalized to β-actin) in normal gastric mucosa (*n* = 30) and cultured gastric cancer cell lines (mean ± SD, *n* = 5). **e** The positive correlation between *ERG* and *Ets-1* transcript levels in gastric cancer tissues (*n* = 81). **f** Kaplan–Meier curves indicating overall (OS) and first progression (FP) survival of gastric cancer patients derived from Kaplan-Meier plotter with low or high expression of *NONO* (cutoff values = 7825.0 and 7797.0), *ERG* (cutoff values = 225.0 and 126.0), or *Ets-1* (cutoff values = 126.0 and 89.0). **g** The mechanisms underlying *pancEts-1*-drived progression of gastric cancer: as a novel lncRNA, *pancEts-1* interacts with NONO to facilitate the physical interaction between NONO and ERG, resulting in transactivation of ERG and transcription of *Ets-1* that are associated with gastric cancer progression. Student’s *t* test analyzed the difference in **c** and **d**. Pearson’s correlation coefficient analysis in **e**. Log-rank test for survival comparison in **f**

To determine the levels of NONO, ERG, and Ets-1 in gastric cancer, fresh tissues and paraffin-embedded sections were collected from 81 primary cases. Immunohistochemical staining indicated that nuclear and cytoplasmic expression of NONO, ERG, and Ets-1 was obviously detected in cancer cells, while their negative or weak expression was noted in normal and precancerous gastric mucosa (Fig. 6a). The expression of NONO and ERG was detected in 61/81 (75.3%) and 53/81 (65.4%) gastric cancer tissues, and higher in cases with local invasion (*P* < 0.001 and *P* < 0.001), lymph node metastasis (*P* < 0.001 and *P* < 0.001), distant metastasis (*P* = 0.004 and *P* < 0.001), or advanced tumor-node-metastasis (TNM) stage (*P* < 0.001 and *P* < 0.001, Supplementary Table S1). Notably, simultaneous expression of NONO and ERG was observed in 48/81 (59.3%) gastric cancer specimens (Supplementary Table S1). The ERG expression was positively correlated with that of Ets-1 in these gastric cancer specimens (*R* = 0.760, *P* < 0.001, Supplementary Table S2). Western blot and real-time qRT-PCR assays revealed the upregulation of *NONO* and *ERG* in gastric cancer specimens, especially in those with metastasis, than that in normal and precancerous gastric mucosa (Fig. 6b, c). Higher *NONO* or *ERG* expression was also detected in cultured gastric cancer cell lines (Fig. 6d). Notably, the expression levels of *ERG* (*R* = 0.649, *P* < 1.0 × 10^−4^) or *NONO* (*R* = 0.644, *P* < 1.0 × 10^−4^) were positively correlated with those of *Ets-1* in gastric cancer specimens (Fig. 6e and Supplementary Figure S3F). In addition, positive expression correlation of *Ets-1* with *pancEts-1* or *ERG* was observed in many types of cancers in public datasets derived from GEO (Supplementary Figure S4 and Figure S5). Kaplan–Meier survival analysis of public gastric cancer datasets derived from Kaplan-Meier plotter indicated significant difference in OS and FP survival of patients with low or high expression of either *NONO* (*P* = 1.0 × 10^−4^ and *P* = 3.0 × 10^−3^), *ERG* (*P* = 1.3 × 10^−7^ and *P* = 1.6 × 10^−5^), or *Ets-1* (*P* = 4.0 × 10^−4^ and *P* = 3.6 × 10^−2^, Fig. 6f). Moreover, specimens with simultaneous high expression of any two or all of *NONO*/*EGR*/*Ets-1* genes were associated with poorer survival possibility of patients, than those with low levels (Supplementary Figure S6). In our series of 81 gastric cancer cases, univariate analysis revealed that the levels of either *pancEts-1* (*P* < 0.001), *NONO* (*P* = 0.003), *ERG* (*P* < 0.001), or *Ets-1* (*P* < 0.001) were respectively associated with poor survival of patients (Supplementary Table S3). However, multivariate analysis indicated that distant metastasis [hazard ratio (HR) = 2.151, *P* = 0.006], but not simultaneous high expression of all *pancEts-1*/*NONO*/*ERG*/*Ets-1* genes (HR = 3.012, *P* = 0.105), was an independent prognostic factor for predicting poor survival of these patients (Supplementary Table S4). These results indicated the expression association of *NONO*, *ERG*, and *Ets-1* with poor outcome of gastric cancer patients.

## Discussion

Since first indentified as the cellular proto-oncogene of retroviral v-ETS [22], a series of studies have demonstrated that Ets-1 regulates transcription through recognizing binding sites within gene promoters, and participates in many biological processes, such as cellular differentiation, proliferation, transformation, angiogenesis, and cancer progression [9]. Ets-1 facilitates cell cycle G1/S progression and cellular proliferation via up-regulating cyclin E and cyclin dependent kinase 2 [23]. During cancer invasion and metastasis, Ets-1 facilitates the expression of urokinase type plasminogen activator and matrix metalloproteinase 9 [24–26]. Human *Ets-1* promoter contains potential binding sites for ETS proteins, specificity protein 1 (Sp1), activator protein 1 (AP1), and AP2 [27]. However, the transcriptional regulators of *Ets-1* in human cancers remain to be determined. In the current study, we identify *pancEts-1* as a lncRNA associated with poor survival of gastric cancer. We demonstrate that *pancEts-1* binds to NONO protein to facilitate the transactivation of ERG and subsequent transcription of *Ets-1* (Fig. 6g). The discovery of such a lncRNA represents a promising step for the therapeutic intervention against gastric cancer. In addition, *pancEts-1* exerts oncogenic functions to drive the tumorigenesis and aggressiveness of gastric cancer cells, suggesting novel mechanisms underlying gastric cancer progression.

NONO is a 54-kDa nuclear and multifunctional RNA-binding and DNA-binding protein that participates in various nuclear events, including transcriptional regulation, RNA processing, and DNA repair [21]. NONO regulates the transcription of rhodopsin [28] and survivin [29], and functions as a coactivator of transcription factors androgen receptor [30], estrogen receptor alpha [31], progesterone receptor [32], thyroid hormone receptor [33], retinoid X receptor [33], and SPI1 [34] to regulate the expression of their respective target genes. Previous studies show that NONO is elevated in many human cancers, including prostate cancer [35], melanoma [36], and neuroblastoma [37]. Meanwhile, knockdown of *NONO* inhibits the proliferation of melanoma cell lines [36] and counteracts the oxaliplatin resistance in colorectal cancer cells [38]. In this study, our data suggested that NONO was associated with poor survival of gastric cancer patients, and NONO facilitated the growth, invasiveness, and metastasis of gastric cancer cells, indicating the oncogenic properties of NONO in gastric cancer progression.

Recent evidence shows that NONO interacts with lncRNAs to regulate gene expression. A nuclear lncRNA *LINC00473* binds to NONO to facilitate gene transcription [39]. In neuroblastoma, NONO binds to *lncUSMycN*, a lncRNA transcribed from approximately 14 kb upstream of *MYCN* TSS, to post-transcriptionally up-regulate the *MYCN* expression [37]. In addition, gastric adenocarcinoma predictive long intergenic noncoding RNA (*GAPLINC*) promotes the invasion of colon cancer cells through interacting with NONO and stimulating the expression of snail family zinc finger 2 [40]. Structurally, NONO protein is consisted of RRM domains, DNA binding domain, and a coiled-coil domain [21]. In this study, we demonstrate that *pancEts-1* binds to the RRM1 domain of NONO protein. Since knockdown of *NONO* rescued the gastric cancer cells from alteration in biological behaviors induced by *pancEts-1*, our evidence suggests that *pancEts-1* exerts oncogenic functions, at least in part, via regulating the NONO activity.

As one transcription factor of Ets protein family, ERG participates in vasculogenesis, angiogenesis, haematopoiesis, and bone development [41]. In human cancers, chromosomal translocation, gene fusion, and amplification of *ERG* locus have been documented in acute myeloid leukemia, prostate cancer, and Ewing’s sarcoma [41]. Enforced expression of *ERG* results in morphological transformation of murine NIH3T3 cells [42], while silencing of *ERG* gene inhibits the proliferation and aggressiveness of prostate cancer cells [41], revealing its oncogenic potential. Previous studies show that ERG regulates the expression of frizzled class receptor 4 [43], E-cadherin [43], vimentin [43], ras homolog family member A [44], vascular endothelial growth factor receptor 2 [45], and zinc finger E-box binding homeobox 1/2 [46] during cancer metastasis and epithelial–mesenchymal transition process. In this study, we demonstrate that ERG serves as a crucial transcriptional regulator that promotes the expression of *Ets-1*, while NONO functions as a coactivator of ERG in gastric cancer. In addition, we identify that *pancEts-1* is essential for the interaction between NONO and ERG, which results in ERG transactivation and *Ets-1* upregulation that are associated with gastric cancer progression.

In summary, for the first time, our data demonstrate that *pancEts-1* is upregulated and associated with poor outcome of gastric cancer. LncRNA *pancEts-1* directly interacts with NONO to increase its interaction with ERG, resulting in transactivation of ERG, increase of *Ets-1* expression, and promotion of the tumorigenesis and aggressiveness of gastric cancer cells. These results reveal the regulatory mechanisms of *Ets-1* expression essential for gastric cancer progression, and suggest that *pancEts-1*/NONO/ERG/Ets-1 axis might be of potential values as a novel target for the treatment of gastric cancer.

## Materials and methods

### Cell culture

Human gastric cancer MKN-45 (JCRB0254), MKN-74 (JCRB0255), NCI-N87 (CRL-5822), and AGS (CRL-1739) cells were purchased from Japanese Collection of Research Bioresources Cell Bank (Osaka, Japan) and American Type Culture Collection (Rockville, MD), authenticated by short tandem repeat (STR) profiling, and applied for study within six months following resuscitation of frozen aliquots. Cancer cells were cultured in RPMI1640 medium supplied with 10% fetal bovine serum (Thermo Fisher Scientific, Inc., Waltham, MA) at 37 °C in a humidified atmosphere of 5% CO_2_.

### Northern blotting

The probe of 138-bp in length was prepared in accordance with the manual of PCR DIG Probe Synthesis Kit (Roche, Indianapolis, IN), using primers shown in Supplementary Table S5. Northern blotting was conducted as previously reported [47].

### RACE assay

To amplify the 5′ and 3′ ends of *pancEts-1*, total RNAs of MKN-45 cells were extracted to prepare cDNA. PCR amplification was undertaken using SMARTer RACE cDNA Amplification Kit (Clontech, Mountain View, CA), gene specific primers (Supplementary Table S5), and universal primer mix.

### RNA-FISH assay

To prepare biotin-labeled sense or antisense RNA probe of *pancEts-1*, the biotin RNA Labeling Mix (Roche) and T7 RNA polymerase were used. Fixed cells were hybridized with sense or antisense probe for 16 h at 37 °C, with or without treatment of RNase A (20 μg). Cells were then treated with streptavidin-conjugated Cy3 and 4′,6-diamidino-2- phenylindole (DAPI).

### Gene over-expression or knockdown

Human *pancEts-1* cDNA (1383 bp), *ERG* cDNA (1461 bp), and their truncations were obtained by PCR from gastric cancer specimens (Supplementary Table S6), and inserted into pcDNA3.1 (Invitrogen, Carlsbad, CA) and pCMV-HA (Beyotime Biotechnology, Haimen, China), respectively. Human *NONO* cDNA (1416 bp) was kindly provided by Dr. Jean-Yves Masson [48], and its truncations were prepared by PCR amplification with primers (Supplementary Table S6) and inserted into pCMV-3Tag-1A (Addgene, Cambridge, MA). Oligonucleotides encoding shRNAs specific for *pancEts-1*, *NONO*, or *ERG* (Supplementary Table S6) were inserted into GV102 (Genechem Co., Ltd, Shanghai, China), with scramble shRNA (sh-Scb) as a control (Supplementary Table S6). After screening with neomycin or puromycin (Invitrogen), stable cancer cells were established.

### Real-time quantitative RT-PCR

Nuclear, cytoplasmic, and total RNAs were prepared with RNA Subcellular Isolation Kit (Active Motif, La Hulpe, Belgium) and RNeasy Mini Kit (Qiagen Inc., Redwood City, CA), respectively. The Transcriptor First Strand cDNA Synthesis Kit (Roche) was applied for reverse transcription. For real-time PCR, SYBR Green PCR Master Mix (Applied Biosystems, Foster City, CA) and primers (Supplementary Table S5) were applied, with transcript levels being determined by 2^-△△Ct^ method.

### Western blot

Protein of cancer cells or tissues was prepared using 1× cell lysis buffer (Promega). Western blotting was performed as previously reported [12, 13, 49–51], with antibodies specific for NONO (ab70335), ERG (ab92513), Ets-1 (ab26096), FLAG (ab45766), HA (ab9110, Abcam Inc., Cambridge, MA), histone H3 (sc-10809), GST (sc-33614), and β-actin (sc-130300, Santa Cruz Biotechnology, Santa Cruz, CA).

### Co-immunoprecipitation

Co-IP assay was performed as reported previously [52], using antibodies for NONO (ab70335), HA (ab32572), or FLAG (ab45766, Abcam Inc.). After releasing from bead-bound complex, protein levels were measured by western blotting.

### Luciferase reporter assay

Human *Ets-1* promoter (−1378/+292 bp) was amplified from genomic DNA with primers (Supplementary Table S6), inserted into pGL3-Basic (Promega), and confirmed by sequencing. The mutant binding sites of ERG was prepared using GeneTailor^TM^ Site-Directed Mutagenesis System (Invitrogen) and PCR primers indicated in Supplementary Table S6. Dual-luciferase assay was undertaken as reported previously [51–55].

### Target gene rescue experiments

To rescue the target gene expression upon *pancEts-1* over-expression, transfection of *NONO* specific shRNA (Supplementary Table S6) into cancer cells was performed according to the manual of Genesilencer Transfection Reagent (Genlantis, San Diego, CA).

### RNA pull-down and mass spectrometry assays

The preparation of biotin-labeled RNA probes for *pancEts-1* truncates were described above. RNA pull-down was performed as reported previously [51]. Western blotting and mass spectrometry assay (Wuhan Institute of Biotechnology, Wuhan, China) were applied to measure the retrieved protein.

### Cross-linking RIP assay

After cross-linkage of cells using ultraviolet light (200 J/cm^2^, 254 nm) [51], Magna RIP^TM^ RNA-Binding Protein Immunoprecipitation Kit (Millipore, Temecula, CA) and NONO antibodies (ab70335, sc-136296, Abcam Inc. and Santa Cruz Biotechnology) were applied for RIP assay. Real-time qRT-PCR or RT-PCR with primers (Supplementary Table S5) were used to measure the co-precipitated RNAs, with isotype IgG and total RNAs as negative or input controls, respectively.

### In vitro binding assay

To generate GST-tagged protein, *NONO* truncates were amplified with PCR primers (Supplementary Table S6), subcloned into pGEX-6P-1 (Addgene), and transformed into *E. coli* [51]. The TranscriptAid T7 High Yield Transcription Kit (Fermentas, Waltham, MA) was used to generate the *pancEts-1* cRNA. The NONO–RNA complexes were recovered by GST beads (Sigma), while western blot and RT-PCR using primers (Supplementary Table S5) were applied for detection of protein and RNA, respectively.

### RNA EMSA assay

The preparation of biotin-labeled *pancEts-1* truncates were described above. Following incubation with nuclear extracts or recombinant NONO protein, the LightShift Chemiluminescent RNA EMSA Kit (Thermo Fisher Scientific, Inc.) was applied for RNA EMSA assay.

### ChIP and real-time qPCR assays

ChIP assay was undertaken using the EZ-ChIP kit (Upstate Biotechnology, Temacula, CA) [52, 54, 56]. For real-time qPCR assay, SYBR Green PCR Master Mix (Applied Biosystems) and gene promoter-specific primers (Supplementary Table S5) were applied.

### Cellular viability, growth, and invasion assays

The MTT (Sigma) colorimetric [52, 53, 57], colony formation [53, 58], soft agar [50, 51, 54] and matrigel invasion [12, 13, 49–51, 56, 58] assays for detecting the viability, growth, and invasiveness of cancer cells were undertaken as previously reported.

### Tumor growth and metastasis assays

The Animal Care Committee of Tongji Medical College approved all animal studies (approval number: Y20080290), which were performed according to NIH Guidelines for the Care and Use of Laboratory Animals. Four-week-old female BALB/c nude mice were randomized blindly (*n* = 5 per group) for subcutaneous xenografts (1 × 10^6^ cancer cells for each mouse) and experimental metastasis (0.4 × 10^6^ cancer cells for each mouse) studies as previously reported [12, 56, 57].

### Clinical tissues

The Institutional Review Board of Tongji Medical College approved all human tissue studies (approval number: 2011-S085). All procedures were performed in accordance with guidelines in the Declaration of Helsinki. Written informed consent was obtained from all patients. Normal gastric mucosa tissues were obtained from 30 cancer-free cases underwent biopsies. Fresh cancer and precancerous specimens from 81 primary cases of gastric cancer were obtained during surgery and stored at −80 °C until use.

### Immunohistochemistry

Immunohistochemical staining was undertaken as reported previously [12, 13, 57, 58], with antibody specific for NONO (ab70335), ERG (ab92513), Ets-1 (ab26096, Abcam Inc.; 1:200 dilutions).

### Statistical analysis

The data were presented as mean ± standard deviation (SD). The cutoff for gene expression was defined by average values. The *χ*^2^ analysis, analysis of variance, and Student’s *t*-test were undertaken for comparing the difference of cancer cells or tissues. Statistical significance of overlap or expression correlation was determined by Fisher exact probability assay and Pearson’s correlation coefficient analysis, respectively. Cox regression and Log-rank assays were respectively used to compare the hazard ratios and survival. Two-sided statistical analysis was applied.

## Electronic supplementary material

Supplementary Figure Legends

Supplementary Figure S1

Supplementary Figure S2

Supplementary Figure S3

Supplementary Figure S4

Supplementary Figure S5

Supplementary Figure S6

Supplementary Table S1

Supplementary Table S2

Supplementary Table S3

Supplementary Table S4

Supplementary Table S5

Supplementary Table S6
