# Supplementary material for: Ets-1 promoter-associated noncoding RNA regulates the NONO/ERG/Ets-1 axis to drive gastric cancer progression
Source: Oncogene. 2018 May 18;37(35):4871–86. doi: 10.1038/s41388-018-0302-4 (PMC6117270; doi:10.1038/s41388-018-0302-4)
Supplement: Supplementary file 1 — Supplementary Figure Legends [file 41388_2018_302_MOESM1_ESM.doc]

**Supplementary Figure Legends**

**Supplementary Figure S1 Characterization of *pancEts-1*.** The 5'- and 3'-RACE (**A**), Northern blot (**B**), and real-time qRT–PCR (**C**, mean ± SD, n=4) showing the identification, existence, and distribution of *pancEts-1* in MKN-45 cells. Mining of ribosome profiling data (**D**) and cBioPortal (**E**, [http://cbioportal.org](http://cbioportal.org/)) indicating the protein-coding potential and genetic abnormality of *pancEts-1*.

**Supplementary Figure S2 Kaplan–Meier survival curves of *pancEts-1* in human cancers.** Mining of public datasets indicating the survival curves of patients with low or high levels of *pancEts-1* in human cancers. Numbers in bracket represent the number of patients in the respective groups. Log-rank test for survival comparison.

**Supplementary Figure S3 Roles of *pancEts-1*, NONO, and ERG in gastric cancer cells.** MTT colorimetry (**A**), RIP (**B**), real-time qRT-PCR (**C**, **E**, **F**), and co-IP (**D**) indicating the viability, NONO interaction with *pancEts-1* or ERG, and expression of *NONO* and *Ets-1* (mean± SD, n=4). **P*<0.01 vs. sh-Scb, mock, or mock+sh-Scb. NS, not significant.

**Supplementary Figure S4 Correlation between *pancEts-1* and *Ets-1* expression in human cancers.** Mining of public datasets derived from GEO indicating the correlation between *pancEts-1* and *Ets-1* expression in breast cancer, cervical cancer, colon cancer, gastric cancer, hepatoblastoma, lung cancer, lymphoma, ovarian cancer, and pancreatic cancer.

**Supplementary Figure S5 Correlation between *ERG* and *Ets-1* expression in human cancers.** Mining of public datasets derived from GEO indicating the correlation between *ERG* and *Ets-1* expression in breast cancer, cervical cancer, colon cancer, gastric cancer, hepatoblastoma, lung cancer, lymphoma, ovarian cancer, and pancreatic cancer.

**Supplementary Figure S6 Kaplan–Meier survival curves of *NONO*, *ERG*, and *Ets-1* in gastric cancer patients.** Kaplan-Meier curves indicating overall (OS) and first progression (FP) survival of patients derived from KM plotter with simultaneous high or low expression of *NONO*, *ERG*, and *Ets-1*. Log-rank test for survival comparison.
