# Supplementary figures and images for: Ets-1 promoter-associated noncoding RNA regulates the NONO/ERG/Ets-1 axis to drive gastric cancer progression
Source: Oncogene. 2018 May 18;37(35):4871–86. doi: 10.1038/s41388-018-0302-4 (PMC6117270; doi:10.1038/s41388-018-0302-4)

# Supplementary Figure S1

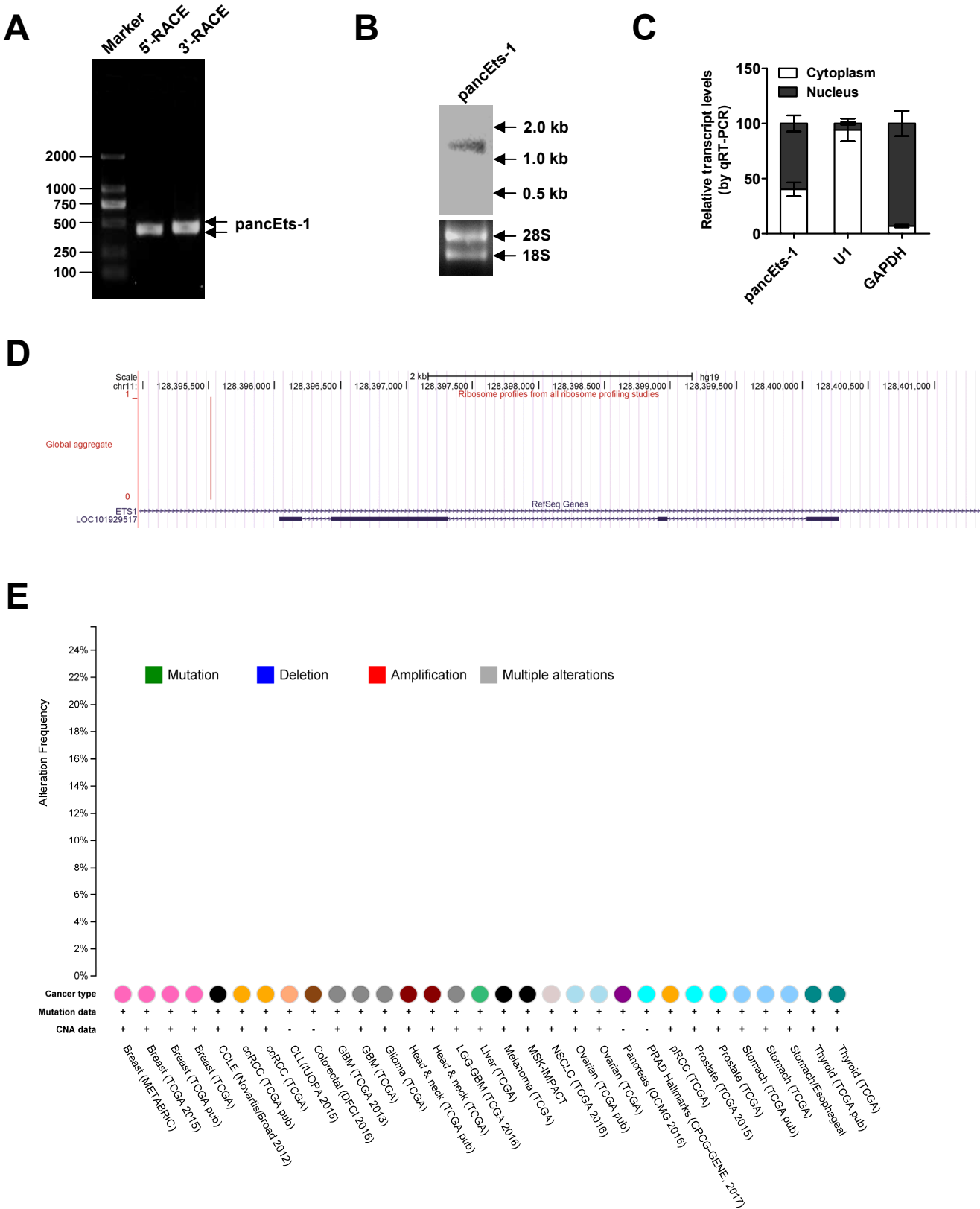

Supplement: Supplementary file 2 — Supplementary Figure S1 [file 41388_2018_302_MOESM2_ESM.pdf]

# Supplementary Figure S2

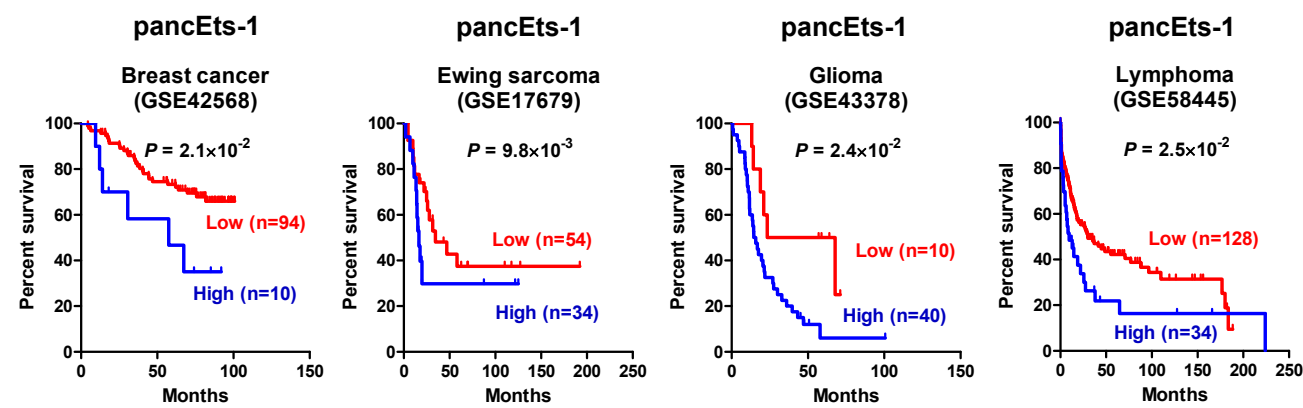

Supplement: Supplementary file 3 — Supplementary Figure S2 [file 41388_2018_302_MOESM3_ESM.pdf]

# Supplementary Figure S3

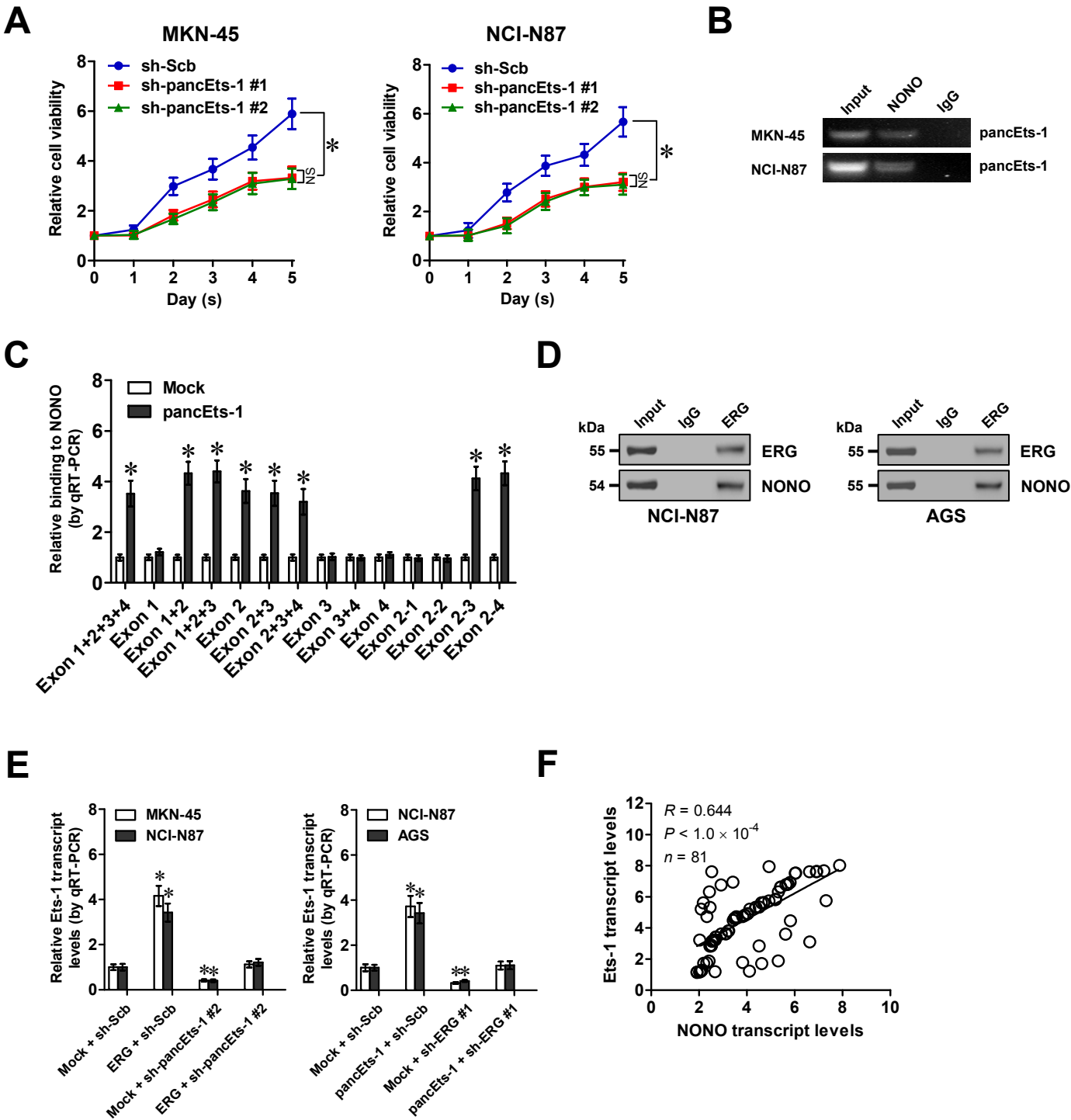

Supplement: Supplementary file 4 — Supplementary Figure S3 [file 41388_2018_302_MOESM4_ESM.pdf]

## Supplementary Figure S4

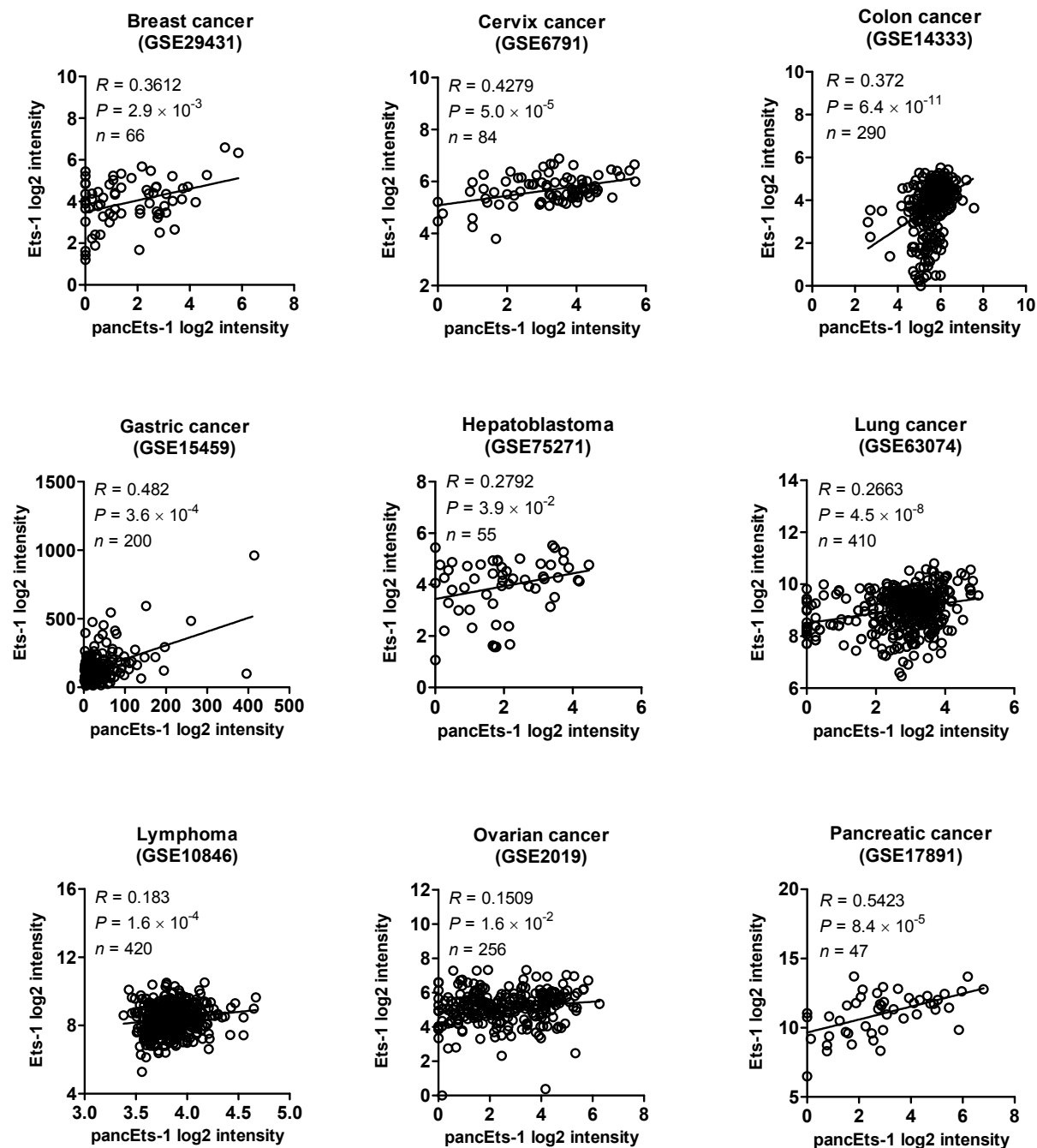

Supplement: Supplementary file 5 — Supplementary Figure S4 [file 41388_2018_302_MOESM5_ESM.pdf]

# Supplementary Figure S5

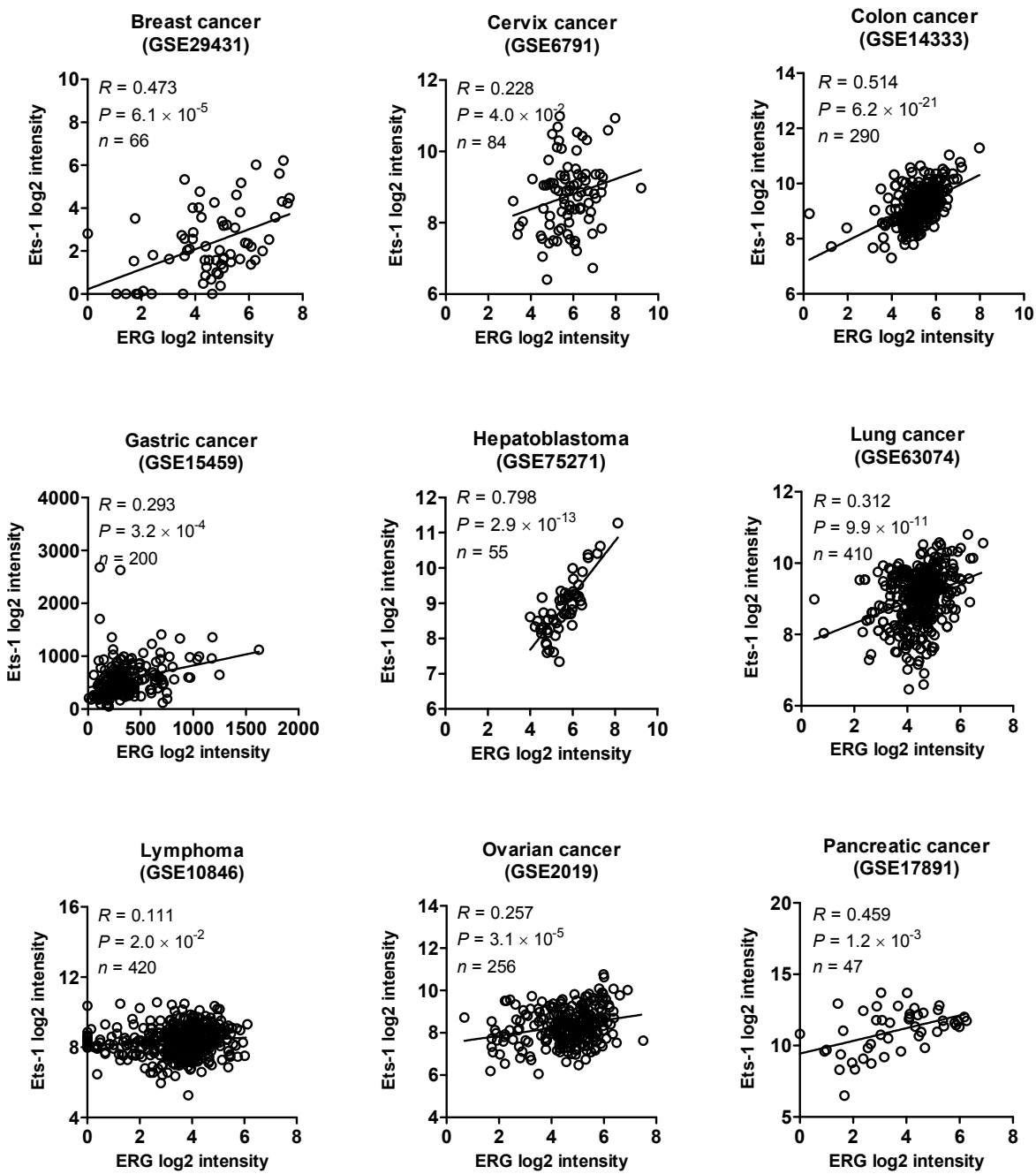

Supplement: Supplementary file 6 — Supplementary Figure S5 [file 41388_2018_302_MOESM6_ESM.pdf]

# Supplementary Figure S6

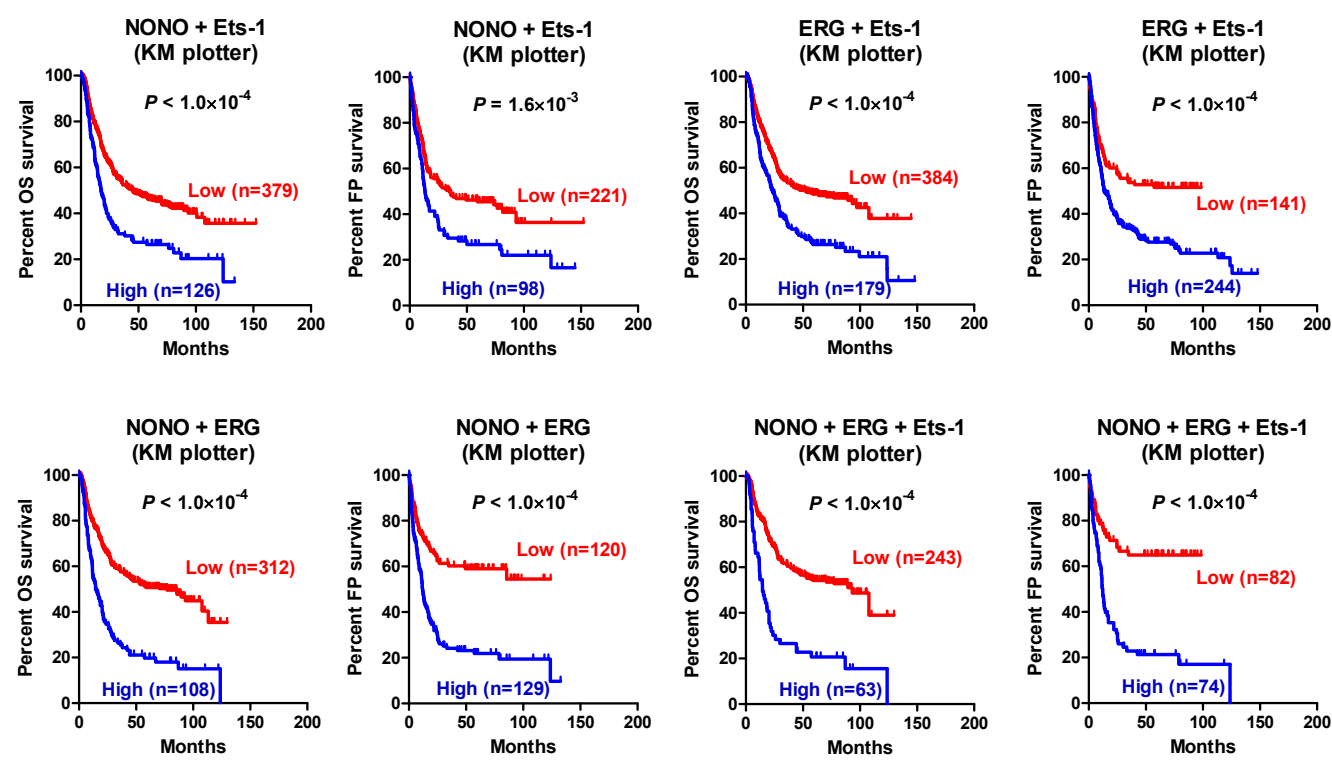

Supplement: Supplementary file 7 — Supplementary Figure S6 [file 41388_2018_302_MOESM7_ESM.pdf]
