# Supplementary material for: Ets-1 promoter-associated noncoding RNA regulates the NONO/ERG/Ets-1 axis to drive gastric cancer progression
Source: Oncogene. 2018 May 18;37(35):4871–86. doi: 10.1038/s41388-018-0302-4 (PMC6117270; doi:10.1038/s41388-018-0302-4)
Supplement: Supplementary file 8 — Supplementary Table S1 [file 41388_2018_302_MOESM8_ESM.doc]

**Supplementary Table S1 NONO and ERG expression in human gastric cancer tissues**

| **Clinicopathologic factor** | **Total** |  | **NONO expression** | |  | **ERG expression** | |  | **NONO+ERG expression** | |
| --- | --- | --- | --- | --- | --- | --- | --- | --- | --- | --- |
|  | **n (%)** |  | **n (%)** | ***P*-value** |  | **n (%)** | ***P*-value** |  | **n (%)** | ***P*-value** |
| **Age (years)** |  |  |  |  |  |  |  |  |  |  |
| ≤60 | 42 (51.9) |  | 29 (69.0) | 0.175 |  | 27 (64.3) | 0.822 |  | 22 (52.4) | 0.191 |
| >60 | 39 (48.1) |  | 32 (82.1) |  |  | 26 (66.7) |  |  | 26 (66.7) |  |
| **Sex** |  |  |  |  |  |  |  |  |  |  |
| Male | 57 (70.4) |  | 44 (77.2) | 0.544 |  | 34 (59.6) | 0.092 |  | 31 (54.4) | 0.169 |
| Female | 24 (29.6) |  | 17 (70.8) |  |  | 19 (79.2) |  |  | 17 (70.8) |  |
| **Size (diameter)** |  |  |  |  |  |  |  |  |  |  |
| ≤6 cm | 53 (65.4) |  | 40 (75.5) | 0.963 |  | 32 (60.4) | 0.188 |  | 27 (50.9) | 0.036 |
| >6 cm | 28 (34.6) |  | 21 (75.0) |  |  | 21 (75.0) |  |  | 21 (75.0) |  |
| **Laurén classification** |  |  |  |  |  |  |  |  |  |  |
| Intestinal type | 47 (58.0) |  | 36 (76.6) | 0.752 |  | 28 (59.6) | 0.192 |  | 23 (48.9) | 0.026 |
| Diffuse type | 34 (42.0) |  | 25 (73.5) |  |  | 25 (73.5) |  |  | 25 (73.5) |  |
| **Gastric wall invasion** |  |  |  |  |  |  |  |  |  |  |
| T1/T2 | 29 (35.8) |  | 13 (44.8) | <0.001 |  | 11 (37.9) | <0.001 |  | 6 (20.7) | <0.001 |
| T3/T4 | 52 (64.2) |  | 48 (92.3) |  |  | 42 (80.8) |  |  | 42 (80.8) |  |
| **LN metastasis** |  |  |  |  |  |  |  |  |  |  |
| Negative | 22 (27.2) |  | 7 (31.8) | <0.001 |  | 4 (18.2) | <0.001 |  | 0 (0.00) | <0.001 |
| Positive | 59 (72.8) |  | 54 (91.5) |  |  | 49 (83.1) |  |  | 48 (81.4) |  |
| **Distant metastasis** |  |  |  |  |  |  |  |  |  |  |
| Negative | 62 (76.5) |  | 42 (67.7) | 0.004 |  | 34 (54.8) | <0.001 |  | 29 (46.8) | <0.001 |
| Positive | 19 (23.5) |  | 19 (100.0) |  |  | 19 (100.0) |  |  | 19 (100.0) |  |
| **TNM stage** |  |  |  |  |  |  |  |  |  |  |
| I/II | 27 (33.3) |  | 10 (37.0) | <0.001 |  | 3 (11.1) | <0.001 |  | 0 (0.00) | <0.001 |
| III/IV | 54 (66.7) |  | 51 (94.4) |  |  | 50 (92.6) |  |  | 48 (88.9) |  |

NONO, non-POU domain containing octamer binding; ERG, Ets related gene; LN, Lymph node; TNM, tumor-node- metastasis.
