# Supplementary material for: Ets-1 promoter-associated noncoding RNA regulates the NONO/ERG/Ets-1 axis to drive gastric cancer progression
Source: Oncogene. 2018 May 18;37(35):4871–86. doi: 10.1038/s41388-018-0302-4 (PMC6117270; doi:10.1038/s41388-018-0302-4)
Supplement: Supplementary file 9 — Supplementary Table S2 [file 41388_2018_302_MOESM9_ESM.doc]

**Supplementary Table S2 Correlation between the expression of ERG and Ets-1**

|  |  | **Ets-1 expression** | | |  |  |  |  |
| --- | --- | --- | --- | --- | --- | --- | --- | --- |
|  |  | Low |  | High |  | *R*-value |  | *P*-value |
| **ERG expression** | |  |  |  |  |  |  |  |
| Low | | 31 |  | 2 |  | 0.760 |  | <0.001 |
|  | |  |  |  |  |  |  |  |
| High | | 8 |  | 40 |  |  |  |  |

Ets-1, v-ets erythroblastosis virus E26 oncogene homolog 1; ERG, Ets related gene; Pearson’s correlation coefficient was applied to determine the expression correlation.
