# Supplementary material for: Ets-1 promoter-associated noncoding RNA regulates the NONO/ERG/Ets-1 axis to drive gastric cancer progression
Source: Oncogene. 2018 May 18;37(35):4871–86. doi: 10.1038/s41388-018-0302-4 (PMC6117270; doi:10.1038/s41388-018-0302-4)
Supplement: Supplementary file 10 — Supplementary Table S3 [file 41388_2018_302_MOESM10_ESM.doc]

**Supplementary Table S3 Univariate analysis of prognostic factors in gastric**

**cancer patients**

| **Clinicopathologic factor** | ***n*** | **Univariate analysis** | |
| --- | --- | --- | --- |
|  |  | Mean ± SEM | *P*-value |
|  |  | (months) |  |
| **Age (years)** |  |  |  |
| ≤60 | 42 | 35.9 ± 4.4 | 0.938 |
| >60 | 39 | 34.9 ± 3.8 |  |
| **Sex** |  |  |  |
| Male | 57 | 38.3 ± 3.6 | 0.172 |
| Female | 24 | 28.7 ± 4.6 |  |
| **Size (diameter)** |  |  |  |
| ≤6 cm | 53 | 37.2 ± 3.8 | 0.611 |
| >6 cm | 28 | 32.3 ± 4.3 |  |
| **Laurén classification** |  |  |  |
| Intestinal type | 47 | 37.7 ± 4.1 | 0.672 |
| Diffuse type | 34 | 32.3 ± 3.9 |  |
| **Gastric wall invasion** |  |  |  |
| T1/T2 | 29 | 56.3 ± 3.7 | <0.001 |
| T3/T4 | 52 | 23.7 ± 3.0 |  |
| **Lymph node metastasis** |  |  |  |
| Negative | 22 | 62.8 ± 1.4 | <0.001 |
| Positive | 59 | 25.1 ± 2.9 |  |
| **Distant metastasis** |  |  |  |
| Negative | 62 | 39.4 ± 3.5 | 0.001 |
| Positive | 19 | 22.7 ± 3.5 |  |
| **TNM stage** |  |  |  |
| I/II | 27 | 63.1 ± 1.1 | <0.001 |
| III/IV | 54 | 21.6 ± 2.8 |  |
| **pancEts-1 expression** |  |  |  |
| Low | 46 | 44.9 ± 3.9 | <0.001 |
| High | 35 | 23.0 ± 3.5 |  |
| **NONO expression** |  |  |  |
| Low | 46 | 43.1 ± 4.0 | 0.003 |
| High | 35 | 25.5 ± 3.7 |  |
| **ERG expression** |  |  |  |
| Low | 33 | 54.2 ± 3.9 | <0.001 |
| High | 48 | 22.5 ± 2.9 |  |
| **Ets-1 expression** |  |  |  |
| Low | 39 | 56.3 ± 2.9 | <0.001 |
| High | 42 | 16.1 ± 2.4 |  |

pancEts-1, Ets-1 promoter-associated noncoding RNA; NONO, non-POU domain containing octamer binding; ERG, Ets related gene; Ets-1, v-ets erythroblastosis virus E26 oncogene homolog 1; TNM, tumor-node- metastasis. Log-rank test was applied for univariate analysis. *n*, number of patients; SEM, standard error of the mean.
