# Supplementary material for: Ets-1 promoter-associated noncoding RNA regulates the NONO/ERG/Ets-1 axis to drive gastric cancer progression
Source: Oncogene. 2018 May 18;37(35):4871–86. doi: 10.1038/s41388-018-0302-4 (PMC6117270; doi:10.1038/s41388-018-0302-4)
Supplement: Supplementary file 11 — Supplementary Table S4 [file 41388_2018_302_MOESM11_ESM.doc]

**Supplementary Table S4 Multivariate analysis of prognostic factors in gastric**

**cancer patients**

| **Clinicopathologic factor** | ***n*** | **Multivariate analysis** | | |
| --- | --- | --- | --- | --- |
|  |  | Hazard ratio | 95% CI | *P*-value |
| **Age (years)** |  |  |  |  |
| ≤60 | 42 | 0.621 | 0.385-1.306 | 0.324 |
| >60 | 39 |  |  |  |
| **Sex** |  |  |  |  |
| Male | 57 | 1.642 | 0.807-3.722 | 0.359 |
| Female | 24 |  |  |  |
| **Size (diameter)** |  |  |  |  |
| ≤6 cm | 53 | 0.813 | 0.312-1.526 | 0.191 |
| >6 cm | 28 |  |  |  |
| **Laurén classification** |  |  |  |  |
| Intestinal type | 47 | 1.442 | 0.812-2.742 | 0.169 |
| Diffuse type | 34 |  |  |  |
| **Gastric wall invasion** |  |  |  |  |
| T1/T2 | 29 | 1.782 | 0.323-6.453 | 0.607 |
| T3/T4 | 52 |  |  |  |
| **Lymph node metastasis** |  |  |  |  |
| Negative | 22 | 1.511 | 1.103-4.513 | 0.316 |
| Positive | 59 |  |  |  |
| **Distant metastasis** |  |  |  |  |
| Negative | 62 | 2.151 | 1.286-5.511 | 0.006 |
| Positive | 19 |  |  |  |
| **TNM stage** |  |  |  |  |
| I/II | 27 | 3.811 | 1.331-8.232 | 0.261 |
| III/IV | 54 |  |  |  |
| **pancEts-1 + NONO + ERG + Ets-1 expression** |  |  |  |  |
| Negative | 22 | 3.012 | 1.365-8.124 | 0.105 |
| Positive | 24 |  |  |  |

pancEts-1, Ets-1 promoter-associated noncoding RNA; NONO, non-POU domain containing octamer binding; ERG, Ets related gene; TNM, tumor-node-metastasis. Cox regression model was applied for multivariate analysis. *n*, number of patients; 95% CI, 95% confidence interval.
