# Supplementary material for: Ets-1 promoter-associated noncoding RNA regulates the NONO/ERG/Ets-1 axis to drive gastric cancer progression
Source: Oncogene. 2018 May 18;37(35):4871–86. doi: 10.1038/s41388-018-0302-4 (PMC6117270; doi:10.1038/s41388-018-0302-4)
Supplement: Supplementary file 12 — Supplementary Table S5 [file 41388_2018_302_MOESM12_ESM.doc]

**Supplementary Table S5 Primer sets used for RACE, qRT-PCR, RIP, probe, and ChIP**

| **Primer set** | **Primers** | **Sequence** | **Product size (bp)** | **Application** |
| --- | --- | --- | --- | --- |
| pancEts-1 | Forward | 5'-GGAAGAGATGGCTGCTCAGA-3' | 223 | qRT-PCR |
|  | Reverse | 5'-GCAGGACCATCCACTACATT-3' |  |  |
| pancEts-1 | GSP1 | 5'-TAGTCTAAAGAAACTGCAGACAGC-3' |  | 3'-RACE |
|  | GSP2 | 5'-TCCAGCACTCCTATTTACCAGAGG-3' |  | 5'-RACE |
|  | NGSP1 | 5'-TCTCTCCTGACAGCCCATGCCAAG-3' |  | 3'-RACE |
|  | NGSP2 | 5'-GTGTCAGTACTAGAACCAGATGCC-3' |  | 5'-RACE |
| pancEts-1 | Forward | 5'-ACACCCTTGAGACACAAGACAGGA-3' | 138 | RIP, probe |
|  | Reverse | 5'-ACCATAGTGTGGTTATCCTGCAAT-3' |  |  |
| Ets-1 | Forward | 5'-GAAGTCGTCACCCCAGACAACA-3' | 254 | qRT-PCR |
|  | Reverse | 5'-TCAGCACGGTCCCGCACATAGT-3' |  |  |
| NONO | Forward | 5'-GCAGGAGATTCGGATGGGTC-3' | 166 | qRT-PCR |
|  | Reverse | 5'-GCGTTCAGTTGTTGGTGGGG-3' |  |  |
| ERG | Forward | 5'-GAGTGGGCGGTGAAAGAATA-3' | 148 | qRT-PCR |
|  | Reverse | 5'-GGAGATGTGAGAGAAGGATG-3' |  |  |
| β-actin | Forward | 5'-ATCTACGAGGGGTATGCC-3' | 227 | qRT-PCR |
|  | Reverse | 5'-TAGCTCTTCTCCAGGGAG-3' |  |  |
| U1 | Forward | 5'-ACTTACCTGGCAGGGGAGATACC-3' | 137 | qRT-PCR |
|  | Reverse | 5'-CCACTACCACAAATTATGCAGTCG-3' |  |  |
| GAPDH | Forward | 5'-AGAAGGCTGGGGCTCATTTG-3' | 258 | qRT-PCR |
|  | Reverse | 5'-AGGGGCCATCCACAGTCTTC-3' |  |  |
| Ets-1 Set 1  (-1262/-1105) | Forward | 5'-CAAAACTTACCAGAGGCAGA-3' | 158 | ChIP |
|  | Reverse | 5'-CCAGGCACTGAGCTCCAT-3' |  |  |
| Ets-1 Set 2  (-863/-724) | Forward | 5'-CGGTCGTGGGAGGGTTGTTAGT-3' | 140 | ChIP |
|  | Reverse | 5'-CAGTGGTTGGGTTGAAGGGGAA-3' |  |  |

pancEts-1, Ets-1 promoter-associated noncoding RNA; Ets-1, v-ets erythroblastosis virus E26 oncogene homolog 1; NONO, non-POU domain containing octamer binding; ERG, Ets related gene; GAPDH, glyceraldehyde 3-phosphate dehydrogenase; RACE, rapid amplification of cDNA ends; RIP, RNA immunoprecipitation; ChIP, chromatin immunoprecipitation; GSP, gene specific primer; NGSP, nested gene specific primer.
