# Supplementary material for: Ets-1 promoter-associated noncoding RNA regulates the NONO/ERG/Ets-1 axis to drive gastric cancer progression
Source: Oncogene. 2018 May 18;37(35):4871–86. doi: 10.1038/s41388-018-0302-4 (PMC6117270; doi:10.1038/s41388-018-0302-4)
Supplement: Supplementary file 13 — Supplementary Table S6 [file 41388_2018_302_MOESM13_ESM.doc]

**Supplementary Table S6 Oligonucleotide sets used for constructs and short hairpin RNAs**

| **Oligo Set** | **Sequences** |
| --- | --- |
| pcDNA3.1-pancEts-1 | 5'-CGCGGATCCGTCAGGAGGGAAGAAGGGAGGGT-3' (sense); |
|  | 5'-CCGCTCGAGCTCTTCTTTTCACCAACTCAACG-3' (antisense) |
| pcDNA3.1-pancEts-1 AS | 5'-CCGCTCGAGGTCAGGAGGGAAGAAGGGAGGGT-3' (sense); |
|  | 5'-CGCGGATCCCTCTTCTTTTCACCAACTCAACG-3' (antisense) |
| pcDNA3.1-pancEts-1  (Exon 1) | 5'-CGCGGATCCGTCAGGAGGGAAGAAGGGAGGGTG-3' (sense); |
|  | 5'-GCCGCTCGAGCTGTGGCAATTCAGCTGGCTAGTT-3' (antisense) |
| pcDNA3.1-pancEts-1  (Exon 1+2) | 5'-CGCGGATCCGTCAGGAGGGAAGAAGGGAGGGTG-3' (sense); |
|  | 5'-GCCGCTCGAGCTCTTCCTGTCTGAGCCATGTTTG-3' (antisense) |
| pcDNA3.1-pancEts-1  (Exon 1+2+3) | 5'-CGCGGATCCGTCAGGAGGGAAGAAGGGAGGGTG-3' (sense); |
|  | 5'-GCCGCTCGAGCTTTGTGGAAGCCAGTGAAA-3' (antisense) |
| pcDNA3.1-pancEts-1  (Exon 2) | 5'-CGCGGATCCCCATCTCTTTGCAGAATAGCTTGG-3' (sense); |
|  | 5'-GCCGCTCGAGCTCTTCCTGTCTGAGCCATGTTTG-3' (antisense) |
| pcDNA3.1-pancEts-1  (Exon 2+3) | 5'-CGCGGATCCCCATCTCTTTGCAGAATAGCTTGG-3' (sense); |
|  | 5'-GCCGCTCGAGCTTTGTGGAAGCCAGTGAAA-3' (antisense) |
| pcDNA3.1-pancEts-1  (Exon 2+3+4) | 5'-CGCGGATCCCCATCTCTTTGCAGAATAGCTTGG-3' (sense); |
|  | 5'-GCCGCTCGAGTTTTCTCTTCTTTTCACCAACTCAAC-3' (antisense) |
| pcDNA3.1-pancEts-1  (Exon 3) | 5'-CGCGGATCCATGGCTGCTCAGAAGCAGTCTTCA-3' (sense); |
|  | 5'-GCCGCTCGAGCTTTGTGGAAGCCAGTGAAA-3' (antisense) |
| pcDNA3.1-pancEts-1  (Exon 3+4) | 5'-CGCGGATCCATGGCTGCTCAGAAGCAGTCTTCA-3' (sense); |
|  | 5'-GCCGCTCGAGTTTTCTCTTCTTTTCACCAACTCAAC-3' (antisense) |
| pcDNA3.1-pancEts-1  (Exon 4) | 5'-CGCGGATCCCGTTACAGCTCACATTTGATTCTC-3' (sense); |
|  | 5'-GCCGCTCGAGTTTTCTCTTCTTTTCACCAACTCAAC-3' (antisense) |
| pcDNA3.1-pancEts-1  (Exon 2-1) | 5'-CGCGGATCCCCATCTCTTTGCAGAATAGCTTGG-3' (sense); |
|  | 5'-GCCGCTCGAGCCTAAGGTTTTGTCTCCAAAGAGG-3' (antisense) |
| pcDNA3.1-pancEts-1  (Exon 2-2) | 5'-CGCGGATCCTCTTGGATTTGTCACATGTTAGAG-3' (sense); |
|  | 5'-GCCGCTCGAGCTGGTACTTCTCCTGCCAAAGTGA-3' (antisense) |
| pcDNA3.1-pancEts-1  (Exon 2-3) | 5'-CGCGGATCCACACCCTTGAGACACAAGACAGGA-3' (sense); |
|  | 5'-GCCGCTCGAGACCATAGTGTGGTTATCCTGCAAT-3' (antisense) |
| pcDNA3.1-pancEts-1  (Exon 2-4) | 5'-CGCGGATCCTCCCTGTGGGAGACAGCTTGGCAT-3' (sense); |
|  | 5'-GCCGCTCGAGCTCTTCCTGTCTGAGCCATGTTTG-3' (antisense) |
| pCMV-3Tag-1A-NONO | 5'-CGCGGATCCATGCAGAGTAATAAAACTTTTAACTTGGAG-3' (sense); |
|  | 5'-GCCGCTCGAGTTAGTATCGGCGACGTTTGTTTGG-3' (antisense) |
| pCMV-3Tag-1A-NONO  (ΔN) | 5'-CGCGGATCCAGCCGTCTTTTTGTGGGAAATCTT-3' (sense); |
|  | 5'-GCCGCTCGAGTTAGTATCGGCGACGTTTGTTTGG-3' (antisense) |
| pCMV-3Tag-1A-NONO  (ΔC) | 5'-CGCGGATCCATGCAGAGTAATAAAACTTTTAACTTGGAG-3' (sense); |
|  | 5'-GCCGCTCGAGGGTTCCCTTGAATCCTTCCTGCTG-3' (antisense) |
| pCMV-3Tag-1A-NONO  (DBHS) | 5'-CGCGGATCCAGCCGTCTTTTTGTGGGAAATCTT-3' (sense); |
|  | 5'-GCCGCTCGAGGGTTCCCTTGAATCCTTCCTGCTG-3' (antisense) |
| pCMV-3Tag-1A-NONO  (DBHS ΔRRM1) | 5'-CGCGGATCCGCATCCCTTACAGTTCGAAACCTT-3' (sense); |
|  | 5'-GCCGCTCGAGGGTTCCCTTGAATCCTTCCTGCTG-3' (antisense) |
| pCMV-3Tag-1A-NONO  (DBHS ΔRRM1+2) | 5'-CGCGGATCCTTAGATGATGAAGAGGGACTTCCA-3' (sense); |
|  | 5'-GCCGCTCGAGGGTTCCCTTGAATCCTTCCTGCTG-3' (antisense) |
| pCMV-3Tag-1A-NONO  (Coiled-Coil) | 5'-CGCGGATCCGCCATGCGCTGGAAGGCACTCATT-3' (sense); |
|  | 5'-GCCGCTCGAGGGTTCCCTTGAATCCTTCCTGCTG-3' (antisense) |
| pGEX-6P-1-NONO | 5'-CGCGGATCCATGCAGAGTAATAAAACTTTTAACTTGGAG-3' (sense); |
|  | 5'-GCCGCTCGAGTTAGTATCGGCGACGTTTGTTTGG-3' (antisense) |
| pGEX-6P-1-NONO  (ΔN) | 5'-CGCGGATCCAGCCGTCTTTTTGTGGGAAATCTT-3' (sense); |
|  | 5'-GCCGCTCGAGTTAGTATCGGCGACGTTTGTTTGG-3' (antisense) |
| pGEX-6P-1-NONO  (ΔC) | 5'-CGCGGATCCATGCAGAGTAATAAAACTTTTAACTTGGAG-3' (sense); |
|  | 5'-GCCGCTCGAGGGTTCCCTTGAATCCTTCCTGCTG-3' (antisense) |
| pGEX-6P-1-NONO  (DBHS) | 5'-CGCGGATCCAGCCGTCTTTTTGTGGGAAATCTT-3' (sense); |
|  | 5'-GCCGCTCGAGGGTTCCCTTGAATCCTTCCTGCTG-3' (antisense) |
| pGEX-6P-1-NONO  (DBHS ΔRRM1) | 5'-CGCGGATCCGCATCCCTTACAGTTCGAAACCTT-3' (sense); |
|  | 5'-GCCGCTCGAGGGTTCCCTTGAATCCTTCCTGCTG-3' (antisense) |
| pGEX-6P-1-NONO  (DBHS ΔRRM1+2) | 5'-CGCGGATCCTTAGATGATGAAGAGGGACTTCCA-3' (sense); |
|  | 5'-GCCGCTCGAGGGTTCCCTTGAATCCTTCCTGCTG-3' (antisense) |
| pGEX-6P-1-NONO  (Coiled-Coil) | 5'-CGCGGATCCGCCATGCGCTGGAAGGCACTCATT-3' (sense); |
|  | 5'-GCCGCTCGAGGGTTCCCTTGAATCCTTCCTGCTG-3' (antisense) |
| pCMV-HA-ERG | 5'-CGGAAGATCTGGATGATTCAGACTGTCCCGGACCCAG-3' (sense); |
|  | 5'-GCCGCTCGAGTTAGTAGTAAGTGCCCAGATGAGAA-3' (antisense) |
| pCMV-HA-ERG  (ΔN) | 5'-CGGAAGATCTGGATGACCACGAACGAGCGCAGAGTTA-3' (sense); |
|  | 5'-GCCGCTCGAGTTAGTAGTAAGTGCCCAGATGAGAA-3' (antisense) |
| pCMV-HA-ERG  (ΔC) | 5'-CGGAAGATCTGGATGATTCAGACTGTCCCGGACCCAG-3' (sense); |
|  | 5'-GCCGCTCGAGGATCCCGTGGAAGTCGAACTTGTAG-3' (antisense) |
| pCMV-HA-ERG  (core) | 5'-CGGAAGATCTGGATGACCACGAACGAGCGCAGAGTTA-3' (sense); |
|  | 5'-GCCGCTCGAGGATCCCGTGGAAGTCGAACTTGTAG-3' (antisense) |
| pCMV-HA-ERG  (core ΔETS) | 5'-CGGAAGATCTGGATGACCACGAACGAGCGCAGAGTTA-3' (sense); |
|  | 5'-GCCGCTCGAGATTTGCAAGGCGGCTACTTGTTGGT-3' (antisense) |
| pCMV-HA-ERG  (core ΔPTN) | 5'-CGGAAGATCTGGTTGACTTCAGATGATGTTGATAAAG-3' (sense); |
|  | 5'-GCCGCTCGAGGATCCCGTGGAAGTCGAACTTGTAG-3' (antisense) |
| pGL3-Ets-1  (-1378/+292) | 5'-CGGGGTACCAGGAGCCACAGATTTTTCTGTTCCTG-3' (sense); |
|  | 5'-GCCGCTCGAGGGATGGTAGCAAGTTTGCAGTTACTG-3' (antisense) |
| pGL3-Ets-1  (Mut 1) | 5'-TTAGAAATCATAGCACATTAAAAATAGCTTTCTCTGAGATTGCTC-3' (sense); |
|  | 5'-ATTTTTAATGTGCTATGATTTCTAAGCACATTTGAACTCCCAGCA-3' (antisense) |
| pGL3-Ets-1  (Mut 2) | 5'-GTCTGTGAAATAGCAGACAAAGCTTTGGCCAATGGTGACATAATT-3' (sense); |
|  | 5'-AAGCTTTGTCTGCTATTTCACAGACTCTCCTCTTTTAGATAAAAC-3' (antisense) |
| sh-Scb | 5'-AGGGATACAAGCATATACCACTCGAGTGGTATATGCTTGTATCCCTC-3' (sense); |
|  | 5'-GAGGGATACAAGCATATACCACTCGAGTGGTATATGCTTGTATCCCT-3' (antisense) |
| sh-pancEts-1 #1 | 5'-GATCCCAGACACAAGACAGGATACAATCTCGAGATTGTATCCTGTCTTGTGTCTT  TTTTGGAT-3' (sense); |
|  | 5'-AGCTATCCAAAAAAGACACAAGACAGGATACAATCTCGAGATTGTATCCTGTCTTG  TGTCTGG-3'(antisense) |
| sh-pancEts-1 #2 | 5'-GATCCCCTCCAGCGAGAGATAGAACAACTCGAGTTGTTCTATCTCTCGCTGGAGTT  TTTGGAT-3'(sense); |
|  | 5'-AGCTATCCAAAAACTCCAGCGAGAGATAGAACAACTCGAGTTGTTCTATCTCTCGC  TGGAGGG-3' (antisense) |
| sh-NONO #1 | 5'-CCGGGCTGCTACAATGGAAGGAATTCTCGAGAATTCCTTCCATTGTAGCAGCTTTTT  G-3' (sense); |
|  | 5'- AATTCAAAAAGCTGCTACAATGGAAGGAATTCTCGAGAATTCCTTCCATTGTAGCAGC -3' (antisense) |
| sh-NONO #2 | 5'-CCGGGCAGGCGAAGTCTTCATTCATCTCGAGATGAATGAAGACTTCGCCTGCTTTT  TG-3'(sense); |
|  | 5'-AATTCAAAAAGCAGGCGAAGTCTTCATTCATCTCGAGATGAATGAAGACTTCGCCT  GC-3' (antisense) |
| sh-ERG #1 | 5'-CCGGCCACCCACAGAAGATGAACTTCTCGAGAAGTTCATCTTCTGTGGGTGGTTTTT  G-3' (sense); |
|  | 5'-AATTCAAAAACCACCCACAGAAGATGAACTTCTCGAGAAGTTCATCTTCTGTGGGTGG -3'(antisense) |
| sh-ERG #2 | 5'-CCGGGATGATGTTGATAAAGCCTTACTCGAGTAAGGCTTTATCAACATCATCTTTTT  G-3' (sense); |
|  | 5'-AATTCAAAAAGATGATGTTGATAAAGCCTTACTCGAGTAAGGCTTTATCAACATCAT  C-3' (antisense) |

pancEts-1, Ets-1 promoter-associated noncoding RNA; Ets-1, v-ets erythroblastosis virus E26 oncogene homolog 1; NONO, non-POU domain containing octamer binding; ERG, Ets related gene; sh-Scb, scramble short hairpin RNAs.
